# Transparency, quality, and statistical consistency of meta-analytic systematic reviews in clinical child and adolescent psychology (2022–2024): study protocol for a meta-review

**DOI:** 10.3389/fpsyg.2025.1535606

**Published:** 2025-07-28

**Authors:** Magdalena Siegel, Selina Fanninger, Julia Riedel, Martina Zemp

**Affiliations:** Department of Clinical and Health Psychology, University of Vienna, Vienna, Austria

**Keywords:** metascience, research synthesis, clinical child and adolescent psychology, reproducibility, meta-review

## Abstract

Meta-analytic systematic reviews are crucial for advancing research and practice in Clinical Child and Adolescent Psychology (CCAP). Despite their importance, there has been no systematic investigation into transparency- and quality-related aspects of these reviews in leading CCAP journals. This study protocol (https://osf.io/qhrau/) proposes a meta-review to assess the transparency, methodological quality, and statistical consistency of recent meta-analytic systematic reviews (2022–2024) published in leading journals from CCAP, aiming to improve future practices in the field. We will include meta-analytic systematic reviews from seven leading journals publishing CCAP-related content between 2022 and 2024 (estimated sample size based on piloting = 60). Eligible systematic reviews need to have conducted a frequentist meta-analysis, define eligible populations as children or adolescents between 0 and 20 years (ideally based on primary study sample mean), may include a clinical psychological or psychotherapeutic intervention, and need to focus on clinical psychological outcomes (no comparators defined). We will search *Web of Science (Core Collection)* by combining journal names (fully indexed within this database) and systematic review-related keywords. Eligible meta-analytic systematic reviews will be assessed for transparency (PRISMA-adaptation; newly developed set of items for CCAP-related content), methodological quality (AMSTAR 2), and statistical consistency (statcheck). Descriptive analyses will include overall and domain-based scores, as well as exploratory analyses assessing associations with transparency-promoting factors on review and journal level. This meta-review can shed light on and enhance the transparency, quality, and statistical consistency within meta-analytic systematic reviews from the field of CCAP. In doing so, it may provide guidance for researchers, reviewers, and editors, while laying the groundwork for future meta-studies in this field.

## 1 Introduction

Meta-analytic systematic reviews are paramount to research and practice within the field of Clinical Child and Adolescent Psychology (CCAP; Bellato et al., 2023; Weisz et al., 2023). Commonly placed at the top of the “evidence pyramid” (Murad et al., 2016), they ideally synthesize evidence for researchers, guide practitioners, and inform policy stakeholders through a systematic collation, critical appraisal, and statistical summary of relevant evidence (Cooper et al., 2019; Siddaway et al., 2019; Borenstein et al., 2021). CCAP is concerned with critical developmental periods—namely infancy, childhood, and adolescence—for the (early) onset (Solmi et al., 2022), prognosis, and treatment (Dekkers et al., 2023; Weisz et al., 2023) of mental disorders. Thus, syntheses within this field are not only highly relevant for public health policy, but ultimately also for children and adolescents affected by mental health burdens as well as their caregivers.

The trust placed in these syntheses by various stakeholders along with scientific developments, such as growing primary evidence, novel meta-analytic methods necessitating data re-analysis, and the growing popularity of meta-meta-analyses (Lakens et al., 2017; Polanin et al., 2020; Nguyen et al., 2022), highlight the need for transparent and methodologically sound meta-analytic systematic reviews. This is particularly relevant for reviews published in prestigious, widely disseminated journal outlets, as these are widely read (and cited) by practitioners and researchers. However, a systematic investigation of transparency and methodological quality-related aspects of meta-analytic systematic reviews within leading CCAP journals is currently lacking. Such an undertaking can best be realized through a meta-scientific approach. Meta-science (or “science of science”) examines the practice of science itself in order to analyze and optimize its processes and methods (Ioannidis et al., 2015; Ioannidis, 2018). Meta-reviews, i.e., methodological overviews of reviews (Biondi-Zoccai, 2016; López-Nicolás et al., 2022) are an important tool in this respect, as they can be used to evaluate the transparency, reproducibility, and quality of systematic reviews in a particular field.

This study protocol outlines our plans for a meta-review to examine the transparency, methodological quality, and consistency of statistical reporting in recent meta-analytic systematic reviews from seven leading journals from the field of CCAP. As a first meta-scientific venture into this field, we aim to provide an impression of current practices (and shortcomings) within these domains, and provide guidance for authors, reviewers, and editors.

### 1.1 Transparency of meta-analytic systematic reviews

A first key factor in assessing the trustworthiness of meta-analytic systematic reviews in CCAP is their transparency. Concerns about transparent reporting and ultimately reproducibility (i.e., the same analysis based on the same data yields the same results; Epskamp, 2019) and replicability (i.e., results at a later time support these conclusions; Epskamp, 2019) have accompanied the field of research synthesis from its inception (e.g., Mulrow, 1987). In part, these concerns sparked the development of the currently ubiquitously used (Caulley et al., 2020) and recommended (Kolaski et al., 2023) PRISMA guidelines (Page et al., 2021a) and their extensions that cover state-of-the-art reporting of protocols (Moher et al., 2015) or search strategies (Rethlefsen et al., 2021) among others. In their current version (March 2021; Page et al., 2021a) the PRISMA guidelines provide detailed guidance on transparent reporting of (meta-analytic) systematic reviews, including on the structure and content of the background information and study aims; the eligibility criteria; systematic search, screening and coding procedures; bias and certainty assessments; meta-analytic procedures; the structure and content of the discussion; conflict of interest statements; protocol preregistration; public availability of materials such as data and code; and using automation tools during the systematic review process (Page et al., 2021a, 2021b). Importantly, PRISMA does not advise on methodological quality (Kolaski et al., 2023). For example, PRISMA recommends reporting the number of authors involved in study screening or coding (Page et al., 2021a), but it does not recommend double coding or interrater reliability thresholds, which indicate sufficient methodological quality (or conduct) within dedicated tools (Shea et al., 2017).

In Psychology and related biopsychosocial disciplines, the ongoing replication crisis (Open Science Collaboration, 2015; Wiggins and Christopherson, 2019) has also increased attention to the transparency and reproducibility of meta-analytic systematic reviews. This has culminated in several meta-scientific investigations within Psychology (Lakens et al., 2017; Hohn et al., 2020; Maassen et al., 2020; Polanin et al., 2020; Steil et al., 2022), Clinical Psychology (López-Nicolás et al., 2022), forensic science (Chin et al., 2022), cognitive training (Sandoval-Lentisco et al., 2024), or biopsychosocial interventions (Page et al., 2018, 2021c; Nguyen et al., 2022) among others. These studies, typically focusing on transparency-related aspects in line with PRISMA, show a relatively consistent pattern: Broad aspects of transparency and those related to narrative sections were most commonly reported, including the background information (Steil et al., 2022), eligibility criteria (Hohn et al., 2020; Polanin et al., 2020; López-Nicolás et al., 2022; Nguyen et al., 2022; Steil et al., 2022; Sandoval-Lentisco et al., 2024), databases searched (Chin et al., 2022; López-Nicolás et al., 2022; Steil et al., 2022; Sandoval-Lentisco et al., 2024) and number of hits retrieved (Nguyen et al., 2022), or the meta-analytic model used (e.g., fixed-effect or random-effects model; Polanin et al., 2020; López-Nicolás et al., 2022; Nguyen et al., 2022; Sandoval-Lentisco et al., 2024). However, detailed information required for reproducibility were often missing, including full and replicable search strategies for all databases searched (López-Nicolás et al., 2022; Nguyen et al., 2022; Steil et al., 2022; Rethlefsen et al., 2024), specific screening and data extraction procedures (Polanin et al., 2020) (but see Sandoval-Lentisco et al., 2024), meta-analytic weighting procedures and estimators of variance components (López-Nicolás et al., 2022; Nguyen et al., 2022; Sandoval-Lentisco et al., 2024), or formulae, information, and raw data to calculate effect sizes (Lakens et al., 2017; Maassen et al., 2020; Polanin et al., 2020; López-Nicolás et al., 2022). Regarding open science practices, the availability of preregistrations or study protocols was rare (Tsujimoto et al., 2017; Polanin et al., 2020; López-Nicolás et al., 2022; Nguyen et al., 2022; Sandoval-Lentisco et al., 2024) and, if existent, deviations from them were often not declared (Tricco et al., 2016; Koensgen et al., 2019; Hu et al., 2021; Sandoval-Lentisco et al., 2025). In a related vein, the sharing of data in interoperable format as well as statistical syntax to reproduce analyses was virtually absent (Polanin et al., 2020; Chin et al., 2022; López-Nicolás et al., 2022; Nguyen et al., 2022; Sandoval-Lentisco et al., 2024).

Currently, it is unclear to what extent these findings also apply to meta-analytic systematic reviews within the field of CCAP. The closest investigation is a meta-review on transparency and reproducibility within meta-analytic systematic reviews of clinical psychological interventions as cited above (López-Nicolás et al., 2022), which concluded that the adherence to these practices was somewhat higher than in other fields (López-Nicolás et al., 2022). Supporting this, three meta-meta-analyses in CCAP, focusing on externalizing behavior problems (Mingebach et al., 2018; Weber et al., 2019) and ADHD (Türk et al., 2023) used a now outdated version of PRISMA guidelines (Moher et al., 2009) as a proxy for study quality (not transparency). They concluded that the adherence to these guidelines was generally adequate (Mingebach et al., 2018; Weber et al., 2019; Türk et al., 2023). However, their scoring approach prevented identifying areas of high or low transparency, and current PRISMA guidelines would allow for a closer examination of finer-grained transparency practices as mentioned above.

Additional transparency considerations may be needed for meta-analytic systematic reviews in CCAP. This notion has been put forward for research syntheses involving pediatric populations, leading to the planned (Kapadia et al., 2016) but, as of November 2024, not yet published PRISMA guideline for such syntheses (PRISMA-C). This guideline is primarily geared toward systematic reviews for pediatric medical intervention research. However, preparatory works related to the development of this guideline (Kapadia et al., 2016; Farid-Kapadia et al., 2017a, 2017b) suggest several reporting domains relevant to CCAP: These include explicitly mentioning the pediatric population in the title, referencing (and justifying) this population within the study objectives, providing replicable age-related eligibility criteria, the reporting of age-specific search terms or tested pediatric search filters, and addressing the implications for children, adolescents, and caregivers specifically within the discussion section (Kapadia et al., 2016; Farid-Kapadia et al., 2017a, 2017b). Given these CCAP-specific considerations, it is crucial to assess their current uptake within meta-analytic systematic reviews in leading journals in the field.

### 1.2 Methodological quality of meta-analytic systematic reviews

A second key factor in assessing the trustworthiness of meta-analytic systematic reviews in CCAP, besides transparency, is their methodological quality (Kolaski et al., 2023). Its assessment (but not the quality itself) necessarily hinges upon transparent and reproducible reporting, but they are not equivalent, as described above. AMSTAR 2 (Shea et al., 2017), the recommended tool within psychology and health-related research (Kolaski et al., 2023), assesses methodological quality within several domains. These include providing a preregistered protocol; the adequacy of the eligibility criteria, literature search, data extraction, and meta-analytic procedures; a duplicate study selection and data extraction procedure; justifying the exclusion of individual studies; conducting risk and publication bias assessments; using appropriate meta-analytic methods, and reporting on potential conflicts of interest within primary studies and the review itself (Shea et al., 2017).

Contrasting the lack of evidence on transparency, the methodological quality of (meta-analytic) systematic reviews in CCAP has been assessed more frequently, likely due to the increased popularity of overviews of reviews in psychology and health-related research that require a quality assessment (Gates et al., 2022). These quality-related assessments as part of overviews of reviews have examined diverse areas of CCAP, such as treatments for anxiety (Byrne et al., 2023; Galán-Luque et al., 2023; Zbukvic et al., 2024), depression (Espada et al., 2023; Zbukvic et al., 2024), eating disorders (García-Fernández et al., 2023), conduct problems (Romero et al., 2023), or consequences of sexual abuse (Sánchez De Ribera et al., 2020); mental health correlates of cyberbullying (Kwan et al., 2020); mental health burdens among sexual and gender minority youth (O’Shea et al., 2024); or the impact of paternal mental health on child development (Scarlett et al., 2024).

Except for one study (Zbukvic et al., 2024), these investigations documented poor methodological quality within most (Kwan et al., 2020; Sánchez De Ribera et al., 2020; Byrne et al., 2023; Romero et al., 2023) or all (Espada et al., 2023; Galán-Luque et al., 2023; García-Fernández et al., 2023; Scarlett et al., 2024; O’Shea et al., 2024) included systematic reviews (notably not all of them meta-analytic). Domains of consistently low quality included the lack of preregistration (Kwan et al., 2020; Sánchez De Ribera et al., 2020; Byrne et al., 2023; Espada et al., 2023; Galán-Luque et al., 2023; García-Fernández et al., 2023; Romero et al., 2023; Scarlett et al., 2024), failure to justify excluding individual studies (Kwan et al., 2020; Sánchez De Ribera et al., 2020; Byrne et al., 2023; Espada et al., 2023; Galán-Luque et al., 2023; García-Fernández et al., 2023; Romero et al., 2023; Scarlett et al., 2024), and no assessment of risk of bias (Kwan et al., 2020; Sánchez De Ribera et al., 2020; Byrne et al., 2023; Galán-Luque et al., 2023; Romero et al., 2023), publication bias (Sánchez De Ribera et al., 2020; Byrne et al., 2023), or primary studies’ funding sources (Kwan et al., 2020; Espada et al., 2023; Romero et al., 2023; Scarlett et al., 2024). These investigations paint a rather bleak picture of the methodological quality of systematic reviews within certain domains and areas of CCAP. They also highlight the need for larger-scaled investigations into this matter (most overviews included fewer than 15 systematic reviews) and clearer guidance on improving methodological quality.

### 1.3 Statistical consistency of meta-analytic systematic reviews

A third key factor in assessing the trustworthiness of meta-analytic systematic reviews is the consistency of statistical reporting within null hypothesis significance testing (NHST; Sterling, 1959). Typically, this involves checking whether reported *p*-values are consistent with their accompanying test statistics and degrees of freedom (Bakker and Wicherts, 2011; Nuijten et al., 2016). Such inconsistencies can indicate various underlying issues, ranging from simple mistakes over misunderstandings of statistics to engaging in questionable research practices, such as incorrectly and deliberately rounding *p*-values below 0.05 (John et al., 2012; Nuijten et al., 2016). Regardless of their cause (and of discussions about whether to abandon NHST), such reporting inconsistencies can lead to false conclusions about the degree to which the data support the presence or absence of an effect (e.g., an effect is erroneously deemed to be significant), hinder reproducibility, and preclude calculating correct effect sizes based on test statistics (Nuijten et al., 2016; Polanin and Nuijten, 2018; Nuijten and Polanin, 2020).

Research has mainly focused on statistical reporting inconsistencies within primary studies (e.g., Bakker and Wicherts, 2011; Nuijten et al., 2016; Olsen et al., 2019; Nuijten and Wicherts, 2024), indicating a disconcertingly high prevalence of statistical reporting errors. For example, a re-analysis of over 250,000 *p*-values from eight major psychology journals (1985–2013) using the R package {statcheck} (Nuijten and Epskamp, 2024) revealed at least one inconsistency between test statistics, degrees of freedom, and *p*-values in about 50% of publications as well as at least one gross inconsistency (i.e., altering conclusions based on statistical significance) in about 13% of publications (Nuijten et al., 2016). Recently, statcheck has been adapted to include test statistics used in meta-analyses (e.g., the *Q* statistic), enabling investigations of meta-analytic systematic reviews (Nuijten and Polanin, 2020).

Some might argue that the primary purpose of a meta-analysis is to provide an estimate of a meta-analytic summary effect (or an exploration of the heterogeneity around this effect), and hence, NHST is of little interest within meta-analytic systematic reviews, including those within CCAP. However, NHSTs in meta-analytic studies are common (previous works documented averages between 29 and 68 NHSTs per article; Polanin and Pigott, 2015; Polanin and Nuijten, 2018) and, for example, routinely used to assess whether the data support the presence or absence of moderator effects, statistical heterogeneity, or, in small samples, even meta-analytic summary effects (Polanin and Pigott, 2015; Nuijten and Polanin, 2020). Furthermore, arguments about inconsistent statistical reporting as litmus tests for deeper underlying issues within a study (Nuijten et al., 2016), as outlined above, similarly apply to meta-analytic investigations (Nuijten and Polanin, 2020). Meta-scientific studies have revealed that 39% of meta-analyses within the social sciences included at least one inconsistent result, whereby 8% contained a gross inconsistency (Polanin and Nuijten, 2018; Nuijten and Polanin, 2020). However, no systematic investigation has yet focused on meta-analytic systematic reviews in CCAP.

### 1.4 The current study

Transparency, quality, and statistical consistency of reporting are crucial for the trust that researchers, clinicians, policymakers, and other stakeholders within the field of CCAP place in meta-analytic systematic reviews. Further, in seeking guidance for future studies, applied meta-analysts within the field of CCAP may want to know in which areas the reporting transparency or quality could be improved to guide their own efforts.

Within this meta-review we aim to answer three research questions (RQs) that all refer to meta-analytic systematic reviews from the field of CCAP within leading journals of this discipline published between 2022 and 2024.

*RQ 1:* How transparent is the reporting of current meta-analytic systematic reviews published within leading journals from the field of CCAP (overall and within broad domains, such as eligibility criteria, systematic search and screening procedure, or effect sizes and statistical synthesis)?

*RQ 2:* How high is the methodological quality of current meta-analytic systematic reviews published within leading journals from the field of CCAP (overall and within domains)?

*RQ 3:* How consistent is the reporting of statistical significance testing in current meta-analytic systematic reviews published within leading journals from the field of CCAP?

As a secondary aim, we plan to gain further insights into transparency-promoting factors by exploring associations between reporting transparency as well as methodological quality, statistical consistency, and other transparency-related variables within our review sample, as meaningful associations between these or related factors have been documented within the literature (Page et al., 2016, 2018; Chin et al., 2022; López-Nicolás et al., 2022; Nguyen et al., 2022). We plan to assess associations between:

- Reporting transparency and methodological quality
- Reporting transparency and statistical consistency
- Reporting transparency and journals’ transparency and openness promotion factor (TOP; Nosek et al., 2015)
- Reporting transparency and self-reported adherence to PRISMA guidelines (yes vs. no)
- Reporting transparency and page limit of journal (no or long page limit vs. short page limit)

As a tertiary aim, we plan to provide practical recommendations for researchers within CCAP by highlighting “what works” (i.e., areas where reporting transparency and methodological quality are high) and “what could work better” (i.e., areas where more transparent reporting or higher quality is advisable for the field), which we will incorporate into our discussion section.

## 2 Methods and analysis

**Figure 1:**
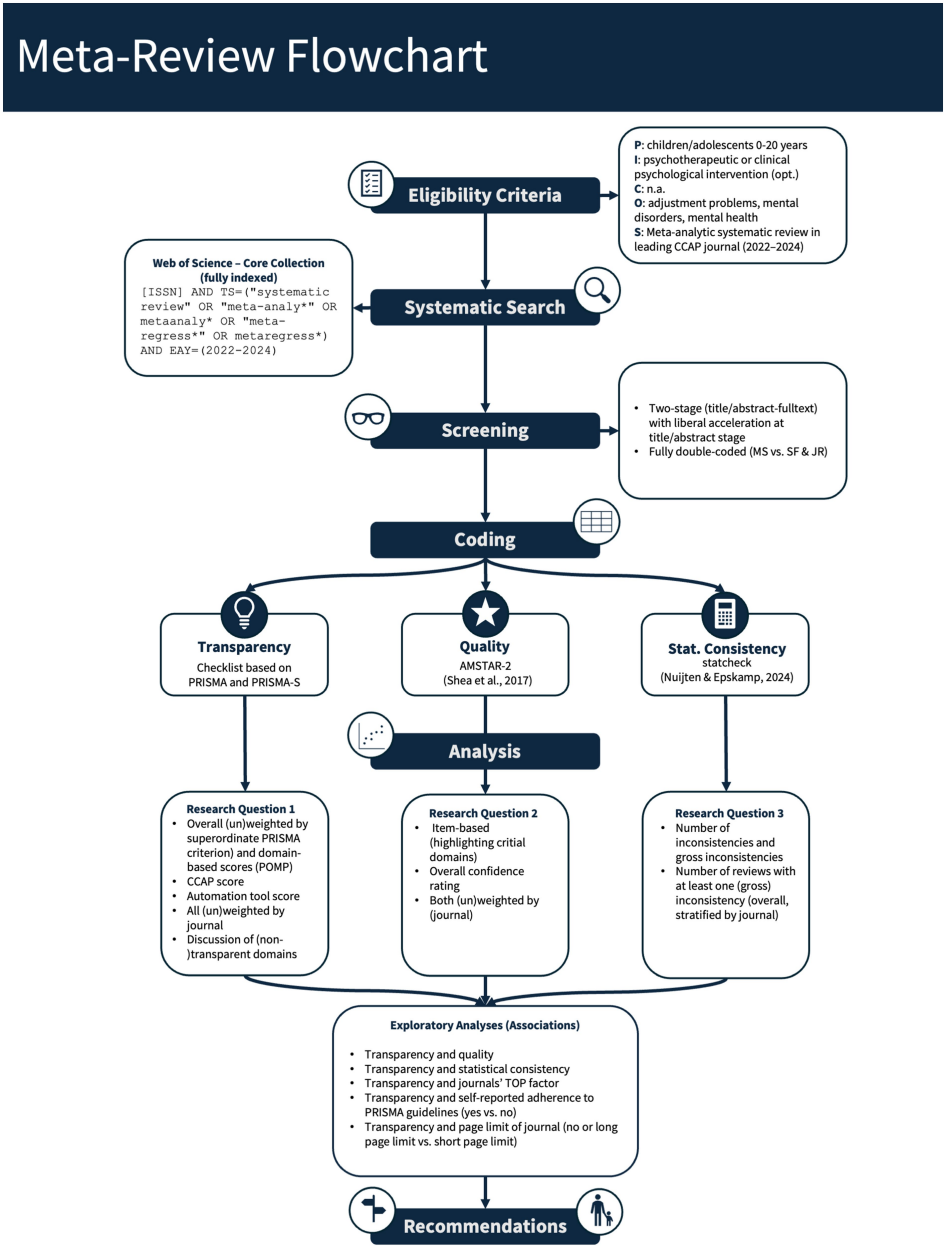
Flowchart of the meta-review.

See Figure 1 for a complete flowchart of this meta-review.

### 2.1 Open science practices, guideline adherence, and transparency statement regarding generative AI

All study and Supplementary materials (S1–S5) for this study protocol can be found in Supplementary material as well as via https://osf.io/qhrau/ (including preprint publication). We will make all data (in machine-readable format) and all materials (including R code to reproduce all analyses, figures, and tables) for the final publication available via this project on OSF, alongside a preprint publication on PsyArXiv. Regarding guideline adherence, there are currently no specific guidelines available for conducting meta-reviews (Dal Santo et al., 2023). Thus, we adhered to the PRIOR statement for overviews of reviews (Gates et al., 2022) together with the PRISMA (Page et al., 2021a), PRISMA-P (Moher et al., 2015) (checklist provided within Supplementary material S1) and PRISMA-S guidelines where applicable (Rethlefsen et al., 2021). The final publication will similarly adhere to these guidelines where applicable. Note that, in contrast to PRIOR, we did not include “overview of reviews” in our title (Gates et al., 2022), as this would imply a content focus rather than a methodological focus inherent to meta-reviews by our definition (Biondi-Zoccai, 2016; López-Nicolás et al., 2022).

During preparation of this manuscript, ChatGPT (versions 3.5 and 4.0; OpenAI, 2024) was used on author-generated text [MS] within narrative sections (Introduction, Discussion) to edit for typography, grammar, clarity, and brevity only, akin to a human co-author (Nakagawa and Lagisz, 2024). Prompts included varieties of the statement “Please edit the following paragraph for brevity and clarity and point out what was corrected: [author-generated text copied in full].” MS incorporated these suggestions as seen fit and, together with the other authors, takes full responsibility for the content of this manuscript. No new text segments, ideas, or references were generated with the help of ChatGPT, any other large language model, or any other generative artificial intelligence assistance.

### 2.2 Conceptual scope regarding the field of CCAP

This meta-review focuses on meta-analytic systematic reviews from the field of CCAP. To translate a definition of this field into precise eligibility criteria, we will first present our definition of CCAP for the purpose of this meta-review and highlight conceptual differences from related disciplines.

In line with the American Psychological Association (Division 53), we define CCAP as a subdiscipline of psychology that examines mental (emotional, behavioral, and developmental) disorders in children and adolescents, including research on the development, maintenance, diagnosis, and clinical-psychological as well as psychotherapeutic interventions/treatment of disorders with a focus on infants, children, and adolescents across all age groups (Brown et al., 2023). We consider developmental psychopathology to be a subarea of CCAP, as it deals with developmental processes that contribute to the etiology, onset, and chronification of mental disorders in children and adolescents, mostly focusing on examining risk and protective factors and trajectories of internalizing and externalizing problems throughout childhood and adolescence in the general population, i.e., including subclinical phenotypes (Barry et al., 2023).

The field of CCAP shares considerable conceptual overlap with Child and Adolescent Psychiatry as well as (Pediatric) Clinical Neuroscience, which are not within the scope of this meta-review. Regarding Child and Adolescent Psychiatry, we base our definition on American Academy of Child and Adolescent Psychiatry (2021) and Schlesinger et al. (2023), which defines it as a medical specialty area that primarily deals with medical aspects of mental disorders and drug/psychopharmacological therapies among children and adolescents. We define the field of (Pediatric) Clinical Neuroscience as an interdisciplinary field combining aspects of neuroscience and clinical medicine: It encompasses the study and treatment of diseases of the nervous system, focusing on the application of neurobiological findings to clinical problems (Kandel et al., 2021) with a typical focus on biological markers, such as genetic (e.g., DNA analyses), neuroimaging (e.g., fMRI), electrophysiological (e.g., EEG), biochemical (e.g., salivary cortisol), and cardiovascular (e.g., heart rate variability) markers.

### 2.3 Eligibility criteria

A concise summary of our eligibility criteria is reported in Table 1. We give additional clarifying information below. We [MS, MZ] conceptualized a first draft of our eligibility criteria based on theoretical and methodological considerations. As recommended (Foo et al., 2021), we then refined these criteria through piloting the screening of a random sample of 55 records (10%) retrieved by our search strategy (see below), as well as through full-text assessments of published meta-analytic systematic reviews from the field of CCAP (Alozkan-Sever et al., 2023; Davis et al., 2023; Manuele et al., 2023; Harris et al., 2024; Lin et al., 2024; Tung et al., 2024; note that these did not need to fulfill final eligibility criteria).

**Table 1:** Eligibility criteria.

Criterion	Inclusion criteria	Notable exclusion criteria
Publication type and language	Published within one of the following journals: *Child Development*, *Clinical Psychology Review*, *Development and Psychopathology*, *European Child & Adolescent Psychiatry*, *Journal of Child Psychology and Psychiatry*, *Journal of the American Academy of Child and Adolescent Psychiatry*, *Psychological Bulletin* Published between 2022 and 2024 (early access date if no volume and issue assigned upon first online publication) Published in English (consequence of journal selection)	Conference abstracts published within these journals Received as first submission prior to March, 2021 due to possible adherence to PRISMA 2009
Study type	Meta-analytic systematic review; i.e., including (i) a clearly formulated research question or aim, (ii) explicit eligibility criteria, (iii) a systematic literature search, (iv) a systematic approach to data extraction, (v) a formal frequentist meta-analysis of at least two effect sizes from two different primary studies.	Systematic reviews evaluating diagnostic test accuracy or psychometric features of an assessment instrument, systematic reviews of qualitative studies Narrative systematic reviews (i.e., without a meta-analysis) Network meta-analyses, Bayesian meta-analyses, individual participant meta-analyses, coordinate-based meta-analyses or other meta-analyses specific to the field of neuroimaging, psychometric meta-analyses, or machine learning-based meta-analyses Other forms of research syntheses, including scoping reviews, rapid reviews, and overviews of reviews
Population	Population of interest (specified within eligibility criteria of the meta-analytic systematic review) are children and/or adolescents between 0 and 20 years (ideally defined as primary study mean age <20 years, see text for fallback criteria)	Adult populations providing retrospective accounts on relevant outcomes within childhood or adolescence.
Intervention	For meta-analytic systematic reviews assessing intervention effects only: assessment of at least one clinical-psychological or psychotherapeutic intervention	For meta-analytic systematic reviews assessing intervention effects only: exclusive assessments of non-psychological interventions, including medical and psychopharmacological
Comparator	–	–
Outcome	Synthesized effect sizes include any kind of child adjustment problems in the domain of (i) externalizing (behavior) and internalizing (emotional) problems, (ii) other mental disorders, or (iii) any indicator of children and adolescents’ overall mental health	–

#### 2.3.1 Publication type and language

We will include meta-analytic systematic reviews (see section ‘Study Type’ for our definition of a meta-analytic systematic review) published as a peer-reviewed article in one of the seven journals as outlined below (see section ‘Information Sources’) within the years 2022 to 2024. We consider the publication year as indexed within *Web of Science* (*Core Collection*; category: early access). Thus, it will be possible that we include articles that were later assigned a volume and issue number in 2025, because their early access publication date was 2024. Note that upon immediate assignment of a volume and issue number, the early access date is identical to this publication date within *Web of Science*. Articles will be excluded if they were received as a first submission at the journal prior to March, 2021, the month were the updated PRISMA guidelines were published, because we deem it unlikely that authors were asked to “update” their guideline adherence from PRISMA 2009 (Liberati et al., 2009) to PRISMA 2020 (Page et al., 2021a) during the review process. From our journal-based inclusion criterion it follows that the publication language will be English (all included journals publish English-language articles exclusively). We will exclude abstracts of conference proceedings published within these journals (e.g., abstracts of the Annual Meeting of the American Academy of Child and Adolescent Psychiatry, as published within the respective journal).

#### 2.3.2 Study type

We will include meta-analytic systematic reviews. Explicit definitions of systematic reviews in overviews of systematic reviews (including meta-reviews) are currently rare and, when reported, poorly described (Faggion and Diaz, 2019; Gates et al., 2022). In our definition of a systematic review, we follow the current PRISMA guidelines that define a systematic review based on the Cochrane Handbook (Higgins et al., 2024) as “a review that uses explicit, systematic methods to collate and synthesize findings of studies that address a clearly formulated research question” (Page et al., 2021a, p. 3). We translated this broad definition into the following explicit eligibility criteria (see Table 1): The publication must (i) present a clearly formulated research question or study aim, (ii) formulate (and follow) explicit eligibility criteria against which potentially eligible studies are assessed (regardless of whether a common scheme, such as PICO, was used), (iii) describe a systematic literature search approach, operationalized as searching at least one (named) database and providing at least examples of keywords used within this search, and (iv) describe a systematic approach to data extraction, with the minimum standard of enumerating or indicating some (key) variables that were extracted.

Systematic reviews that rely on a non-traditional, but still systematic literature search approach (e.g., a forward search of a key reference instead of a keyword search) will be judged on a case-by-case basis within the research team. Decisions regarding eligibility will be transparently reported and systematically applied to all further, similar cases. We consciously kept these eligibility criteria as broad as possible to not systematically exclude systematic reviews of low quality. We will exclude the following study types: Scoping reviews (as defined within Tricco et al., 2018); overviews of (systematic) reviews (Gates et al., 2022), including studies labeled as umbrella reviews, meta-meta-analyses, meta-reviews, or second-order meta-analyses; and systematic reviews evaluating diagnostic test accuracy or psychometric features of an assessment instrument, the latter due to differing reporting guidelines.

Because our aim is to analyze the reporting characteristics of *meta-analytic* systematic reviews, the systematic review also (v) needs to report on a frequentist meta-analysis of effect sizes of at least two primary studies based on the systematic literature search. Here, we again follow the PRISMA guidelines and define a meta-analysis as a “statistical technique used to synthesize results when study effect estimates and their variances are available, yielding a quantitative summary of results” (Page et al., 2021a, p. 3). This broad definition of a meta-analysis translates into the following eligibility criterion: Use of a fixed-effect or random-effects meta-analytic model within a frequentist framework (as outlined, for example, in Borenstein et al., 2021), where models with several variance components (to account for nesting of effect sizes within studies, most commonly a three-level meta-analysis, e.g., Assink and Wibbelink, 2016; Pustejovsky and Tipton, 2022) will be included. We do not place any restriction on the type of meta-analytic effect size or whether moderator analyses were conducted. Following argumentation within previous meta-reviews with a similar aim (López-Nicolás et al., 2022; Sandoval-Lentisco et al., 2024), we will exclude systematic reviews that apply network meta-analyses, Bayesian meta-analyses, individual participant meta-analyses, coordinate-based meta-analyses or other meta-analyses specific to the field of neuroimaging, psychometric meta-analyses, or machine learning-based meta-analyses because these types of meta-analyses require different criteria to assess their quality and transparency of reporting and/or they do not require reporting of statistical information as evaluated within statcheck (Nuijten and Polanin, 2020; Nuijten and Epskamp, 2024), the tool we use to assess statistical reporting consistency.

#### 2.3.3 Population

We will include meta-analytic systematic reviews that define their population of interest within their eligibility criteria as children and adolescents between 0 and 20 years, following World Health Organization (2024). Ideally, this would entail a definition of the mean sample age of <20 years for primary studies (included in the meta-analytic systematic review) as specified within the meta-analytic systematic reviews’ eligibility criteria. If a more ambiguous criterion is reported within these meta-analytic systematic reviews, such as “(…) included at least one measure of offspring externalizing behavior assessed during childhood or adolescence [defined as ages 2–20]” (Tung et al., 2024, p. 6; 18 as upper age limit replaced with 20 for the sake of the example given here), where it is unclear whether this refers to a measure of central tendency or range, we will include the meta-analytic systematic review in case of at least 90% of primary studies with a mean age <20 years. We will proceed the same way in cases where no clear age-related eligibility criteria are defined but it is evident that the meta-analytic systematic review focuses on children or adolescents. We will tally this manually from information within the meta-analytic systematic review if reported (without reviewing primary studies of the respective meta-analytic systematic review by ourselves). We will exclude meta-analytic systematic reviews based on retrospective accounts, i.e., adults reporting on eligible outcomes within childhood or adolescence. If this information (i.e., primary studies’ mean sample age) is not reported within the meta-analytic systematic review, but the (ambiguous) age range is within 0–20 years, we will include the systematic review (without reviewing the primary studies). If the systematic review specifies the population of interest as “children or adolescents” without reporting on age-related criteria or the population of interest is ambiguously specified (e.g., “youth”), we will decide on inclusion on a case-by-case basis within the research team and report the decision for inclusion or exclusion transparently. The implemented approach will be systematically applied to all further, similar cases.

If the systematic review includes both children/adolescent and adult samples, we will include the systematic review in case of at least 90% of primary studies with a mean age <20 years. If this information is not reported, we will exclude the systematic review (without reviewing the primary studies). Note that this eligibility criterion does not imply that self-report data for this population need to be included within the meta-analytic systematic review (see section ‘Outcomes’).

#### 2.3.4 Intervention

We will include both meta-analytic systematic reviews of studies without examining treatments/interventions (e.g., epidemiological and prevalence studies, studies focusing on developmental psychopathology) as well as intervention studies. For meta-analytic systematic reviews that do not synthesize information on intervention effects, this eligibility criterion does not apply. For meta-analytic systematic reviews that synthesize information on intervention effects, we will include those that assess the effect of at least one clinical-psychological or psychotherapeutic intervention within the conceptual scope of CCAP (see section ‘Conceptual Scope Regarding the Field of CCAP’). We do not place any restriction on the recipient of the intervention (e.g., children, caregivers), but the outcome must be assessed for children or adolescents (through any form of rating or assessment, including, e.g., self-reports, other-reports, or clinical assessments). Systematic reviews that only assess effects of non-psychological treatments (e.g., psychopharmacological or other medical interventions) will be excluded, as these would belong to the field of psychiatry or pediatrics. Meta-analytic systematic reviews that include both clinical-psychological or psychotherapeutic interventions together with psychopharmacological interventions will be included.

#### 2.3.5 Comparator

No eligibility criteria defined.

#### 2.3.6 Outcome

Eligible for inclusion are meta-analytic systematic reviews where synthesized effect sizes (and thus, at least one meta-analytic summary effect reported) are based on any kind of child adjustment problems in the domain of externalizing (behavior) and internalizing (emotional) problems, other mental disorders, or any indicator of children and adolescents’ overall mental health. First, internalizing and externalizing problems are defined either as a (1) subclinical phenomenon (for internalizing problems: e.g., anxiety symptoms, depressive symptoms, or a sum score of internalizing problems; for externalizing problems: e.g., noncompliance, hyperactivity, aggressive and disruptive behaviors, delinquency, or a sum score of externalizing problems) or as a (2) clinical diagnosis (e.g., anxiety disorders, depressive disorders, conduct disorders [CD], oppositional defiant disorders [ODD], attention-deficit/hyperactivity disorders [ADHD]) based on the ICD-10 or ICD-11 (World Health Organization, 1993, 2019), or on the DSM-IV or DSM-5 criteria (American Psychiatric Association, 1994, 2013). We will also include outcomes indicating a combination of externalizing and internalizing problems.

Second, synthesized effect sizes related to symptoms or syndromes of other mental disorders included in the two latest revisions of the DSM and ICD (chapter F for ICD-10 or section 06 for ICD-11, respectively) that cannot be clearly categorized as internalizing and externalizing problems will be included (e.g., posttraumatic stress disorder [PTSD], autism spectrum disorders [ASD], obsessive-compulsive disorders [OCD], substance-related and addictive disorders, bipolar disorders, eating disorders, schizophrenia spectrum and other psychotic disorders). They could be assessed as clinical diagnoses based on the above-mentioned classification manuals or as continuous measures of selected symptoms related to these disorders (e.g., in disorder-specific questionnaires or scales).

Third, overall mental health includes any global indicators of psychological distress, level of functioning (global assessment or in different areas of life), perceived (chronic) stress, and indicators of a wide range of (non-specific) psychological symptoms (e.g., self-injurious behavior, suicidal ideation/thoughts, behaviors or attempts) and their severity. We will base our inclusion decision on (1) relevant descriptions within meta-analytic systematic reviews’ eligibility criteria, or, in case it is not sufficiently specified, based on (2) descriptions of measures used within primary studies (if reported within the meta-analytic systematic review). If it is unclear based on (1) and (2), the systematic review will be excluded.

Meta-analytic systematic reviews exclusively synthesizing effect sizes based on child adjustment problems in other domains, such as i.e., academic functioning, physical health chronic diseases, coping skills, social support, life satisfaction, and overall or health-related quality of life will be excluded, as they do not fit into our specific outcome categories defined above. Similarly, as a consequence of our considerations outlined above (see section ‘Conceptual Scope Regarding the Field of CCAP’), we will exclude systematic reviews where effect sizes are exclusively based on biological outcome markers, such as genetic markers (e.g., DNA analyses), neuroimaging markers (e.g., fMRI), electrophysiological markers (e.g., EEG), biochemical marker (e.g., salivary cortisol), and cardiovascular marker (e.g., heart rate variability).

### 2.4 Information sources

We focus on meta-analytic systematic reviews from leading journals (based on content and citation metrics criteria) within the field of CCAP to examine reporting practices of the (potentially) most impactful publications within this field. This may represent an upper threshold of the uptake of good reporting practices, but substantial reporting non-transparency has also been identified within top-ranking journals in Psychological Science (e.g., Polanin et al., 2020).

This is the first (notably unfunded) investigation into transparency, methodological quality, and statistical consistency of reporting within meta-analytic systematic reviews within CCAP. Thus, we deem this approach reasonable to gain a first impression of the field and deduce practical recommendations for researchers, as well as to lay the foundation for future works within this field (e.g., meta-reviews assessing time trends or prevalence estimates on reporting practices within the field). Since there is not one single, non-invitational flagship journal dedicated to publishing systematic reviews within CCAP that we could sample from (a commonly used approach within other psychological disciplines, e.g., Polanin et al., 2020; Siegel et al., 2022), and we deemed a keyword search as too broad (and therefore impractical, if not impossible) to identify eligible systematic reviews, we identified eligible journals through a three-step procedure based on expert opinion and journal metrics, as detailed in Supplementary material S2.

Ultimately, this yielded a list of seven journals we considered to be representative of the top-ranking journals publishing meta-analytic systematic reviews within the field of CCAP (in alphabetical order): *Child Development*, *Clinical Psychology Review*, *Development and Psychopathology*, *European Child & Adolescent Psychiatry*, *Journal of Child Psychology and Psychiatry*, *Journal of the American Academy of Child and Adolescent Psychiatry*, and *Psychological Bulletin*. Together, these journals cover a broad spectrum of clinical topics and areas of application relevant to CCAP and encompass all age groups specified in our eligibility criteria, i.e., from infancy to adolescence/young adults. They publish widely disseminated research syntheses (and primary studies) from around the world using a diverse range of methods and are highly relevant to an international audience (with a broad ethnic background), both in terms of readership and authorship. All journals are indexed in *Web of Science* (*Core Collection*, as subscribed by the University of Vienna). Thus, we rely on this database as our single information source.

### 2.5 Search strategy

Our full search string for *Web of Science* (*Core Collection*, as subscribed by the University of Vienna) is reported in Table 2. We combined (1) all seven unique journal ISSNs (see also Chin et al., 2022, for a similar approach and Table 3 for journal ISSNs) with (2) keywords relating to systematic reviews or meta-analyses (appearing in titles, abstracts, or keywords). These records were then (3) limited to publication years (early access) 2022, 2023, or 2024. Within sets (1) and (2), search terms were combined using the OR-operator, whereas sets (1), (2), and (3) were combined using the AND-operator. No other filters or limits were used. The search string was constructed by the first author [MS] and peer-reviewed for accuracy by the second author [SF]. We did not validate the search string against a pre-specified set of studies, because the keywords used within our search were limited and eligible articles largely defined through their publishing outlets. On September 25, 2024, this search strategy yielded 550 records. After peer review for this protocol has been concluded, we will re-run this search. If peer review has been completed prior to 2025, we will update our search after January 1, 2025, for records from 2024.

**Table 2:** Search string within Web of Science (Core Collection).

Set	Content
1	IS = (“0009–3920” OR “0272–7358” OR “0954–5794” OR “1018–8827” OR “0021–9630” OR “0890–8567” OR “0033–2909”)
2	TS = (“systematic review” OR “meta-analy*” OR metaanaly* OR “meta-regress*” OR metaregress*)
3	EAY = (2022–2024)
4	1 AND 2 AND 3

Web of Science Core Collection as subscribed by the University of Vienna, including Science Citation Index Expanded (SCI-EXPANDED)—1900-present; Social Sciences Citation Index (SSCI)—1900-present; Arts & Humanities Citation Index (AHCI)—1975-present; Conference Proceedings Citation Index – Science (CPCI-S)—1990-present; Conference Proceedings Citation Index – Social Science & Humanities (CPCI-SSH)—1990-present; Book Citation Index – Science; (BKCI-S)—2005-present; Book Citation Index – Social Sciences & Humanities (BKCI-SSH)—2005-present; Emerging Sources Citation Index; (ESCI)—2005-present; Current Chemical Reactions; (CCR-EXPANDED)—1985-present; Index Chemicus (IC)—1993-present. “Exact search” feature of Web of Science turned on. Search performed on September 25, 2024, 550 hits.

**Table 3:** Characteristics of included journals.

Journal	IF22	IF23	TOP	APA format	PRISMA adherence	Page limit	ISSN
Child Development	4.6	3.9	7	Yes	No	Long (50 pages)	0009-3920
Clinical Psychology Review	12.8	13.7	1	No	No	Long (50 pages)	0272-7358
Development and Psychopathology	3.3	3.1	0	Yes	Yes	None	0954-5794
European Child & Adolescent Psychiatry	6.4	6.0	4	No	Yes	Long (12,000 words)	1018-8827
Journal of Child Psychology and Psychiatry	6.5	7.6	5	Yes	Yes	Short (5,000 words)	0021-9630
Journal of the American Academy of Child and Adolescent Psychiatry	13.3	9.2	5	No	Yes	Short (5,000 words)	0890-8567
Psychological Bulletin	22.4	17.3	15	Yes	Yes	None	0033-2909

IF = Impact Factor (Clarivate Analytics). ISSN = International Standard Serial Number (Print). APA-formatting, PRISMA adherence, and page limit assessed through author guidelines from journal websites (August 26, 2024). TOP = Transparency and Openness Promotion Score, assessed through https://topfactor.org/ (August 24, 2024). Page-word conversion as per standard APA formatting recommendation (12 pt, double-spaced) calculated by assuming 250 words/page.

To derive search terms for set (2), we screened previous meta-reviews (Koffel and Rethlefsen, 2016; Cortese et al., 2019; Wayant et al., 2019; Hohn et al., 2020; Chow et al., 2021; Chin et al., 2022; López-Nicolás et al., 2022; Nelson et al., 2022; Nguyen et al., 2022; Steil et al., 2022; Wong and Bouchard, 2023; Pellegrini et al., 2024; Rethlefsen et al., 2024; Sandoval-Lentisco et al., 2024) for keywords and deemed variants of “systematic review” and “meta-analysis” useful for the purpose of this study. We supplemented these with variants of the term “meta-regression” to also include meta-analytic systematic reviews that primarily focused on exploring heterogeneity within effect sizes. We piloted including the term “quantitative review” (as used, for example, in López-Nicolás et al., 2022) as well as “research synthes*” (own considerations), which yielded no additional records and were dropped.

We deliberately chose to search within titles, abstracts, and keywords (vs. a title-only search) and to include variants of the keyword “meta-analysis” (vs. “systematic review” only) because we wanted to avoid limiting our search to those meta-analytic systematic reviews that fulfill the first PRISMA—and thus transparency—criterion, namely to include the term “systematic review” within the title (Page et al., 2021a). Our journal- and study type-based search strategy will also retrieve systematic reviews unrelated to children and adolescents (e.g., from general clinical psychology journals such as *Clinical Psychology Review*) and/or clinical psychology (e.g., from developmental journals such as *Child Development* or multidisciplinary journals such as the *Psychological Bulletin*). We will screen out these reviews manually (see below), a strategy that is feasible given the moderate number of records.

### 2.6 Selection process and expected sample size

We opt for a classic two-stage screening procedure (Page et al., 2021a), including screening of titles and abstracts, followed by full-text screening. We piloted this approach based on the same sample of 55 records that was also used to pilot our eligibility criteria. Title and abstract screening will be undertaken by three authors [MS, SF, JR], who will assess titles and abstracts of every record independently. Records will be advanced to the full-text stage via liberal acceleration (Page et al., 2018), i.e., every record deemed as eligible for full-text assessment by one (or more) author(s) will be assessed as a full-text. For descriptive purposes, we will report our interrater reliability (percentage agreement) between all three authors and between pairs of authors.

Full-text assessment will also be undertaken by [MS, SF, JR], in such that we will assess full-texts’ final eligibility prior to data extraction. Final eligibility will be based on consensus among all three authors. The senior author [MZ] will arbitrate in cases where no consensus can be reached or additional expertise is needed. For descriptive purposes (our inclusion decision is consensus-based in all cases, Shea et al., 2017), we will report initial interrater reliability (percentage agreement between all three authors and pairs of authors). References of records that could not be retrieved in full or that were excluded at this stage will be published together with the reason for their exclusion. For inclusion decisions that we made on a case-by-case basis, we will provide a separate table (possibly within Supplementary material) including a detailed reasoning for each case. The number of excluded records by category will also be made available within the PRISMA flow chart.

For both steps, we will not use any automation tools or rely on crowdsourcing or previously “known” assessments (Page et al., 2021a). We do not expect needing to translate abstracts or articles because of our journal-based search strategy, including English-language articles only. We also do not expect having to contact authors for information necessary to determine eligibility. We will publish a PRISMA flow chart.

We estimate that our final sample of eligible meta-analytic systematic reviews will include about 60 reviews. We base this estimate on our piloting procedure, where we [MS, SF] piloted our eligibility criteria as well as our systematic search procedure, as recommended (Foo et al., 2021). Based on a random sample of 55 out of 550 retrieved records (10%; September 25, 2024), we deemed six as eligible for inclusion, thus totaling 6 * 10 = 60 studies based on extrapolation.

### 2.7 Data collection process

Data collection for research questions (RQ 1; transparency) and (RQ 2; quality) will be undertaken by [MS, SF, JR]. We will randomly split eligible records into two groups. The first author [MS] will code all records once and the second authors [SF, JR] will each code records from one of the two groups. Thus, every eligible record will be coded twice and independently. We will resolve discrepancies through discussion and/or arbitration by another author (SF and JR for records they did not code; MZ for all records). We piloted our data collection process as well as our data collection sheets (see Section ‘Data Items’) on two meta-analytic systematic reviews that were deemed eligible within piloting of the screening procedure (England-Mason et al., 2023; Farkas et al., 2024). In addition, we will code three meta-analytic reviews jointly to reach a common understanding of the codebook and coding materials, in particular regarding quality- and transparency-related items. Through regular meetings between the raters regarding overarching coding questions, we will further strive to ensure a common understanding of the codebook and minimize systematic rater bias or discrepancies.

We will assess and report interrater reliability for every variable (percentage agreement as recommended within AMSTAR-2; Shea et al., 2017) and report on the mean (*M*), standard deviation (*SD*), and range across all variables. Because our assessment of transparency-related variables is based on a self-developed, PRISMA-based checklist (see section 2.8.1.2), we will also report the prevalence-adjusted, bias-adjusted Kappa (PABAK; as calculated within Stevenson and Sergeant, 2025) for all categorical transparency-related variables and pairs of raters and the intraclass correlation (ICC) for continuous variables (as calculated within Gamer et al., 2019). We will not contact authors for additional information, because we are interested in the transparency and quality of reporting as is. We will not use any automation tools for data collection regarding these two research questions.

Data collection for research question 3 (RQ 3; consistency of statistical reporting) will be carried out through statcheck (Nuijten and Polanin, 2020; Nuijten and Epskamp, 2024). Specifically, we will rely on the CRAN version at first date of analysis for the R package {statcheck} (Nuijten and Epskamp, 2024). Statcheck assesses whether reported *p*-values are consistent with their accompanying test statistics by recalculating them (Nuijten and Polanin, 2020). As basis for further analysis via statcheck, we will download .html (preferable) or .pdf-versions of all eligible studies within our meta-review, which will then be read into R and analyzed through functions provided within the package (see section ‘Analysis’). Statcheck can only reliably detect reporting inconsistencies within (correctly) APA-formatted texts (Nuijten and Epskamp, 2024). At time of writing, four out of seven journals included within our meta-review require APA-formatting (Table 3), although this requirement often refers to formatting of references specifically (and not test statistics). We will still include all articles in our analysis (i.e., we will attempt to analyze all downloaded articles through statcheck functions), because it is possible that test statistics from other journals might be formatted in a way that is consistent with APA style and thus analyzable. However, it could be possible that we can only report on results from four journals.

### 2.8 Data items

We will code three groups of items and generate a fourth group of items via statcheck: (1) items related to general publication and study information, (2) transparency-related items (necessary for RQ 1), and (3) quality-related items (necessary for RQ 2) in the form of the AMSTAR 2 tool for assessing methodological quality (Shea et al., 2017). The full codebook is available as a Word document within Supplementary material S3, and as the raw coding sheet (Excel; excepting AMSTAR 2 items which will be coded verbatim within a separate sheet not included for copyright reasons) within Supplementary material S4. We will also analyze items from group (4), which are not manually coded but generated through statcheck.

#### 2.8.1 Item content and development

##### 2.8.1.1 Items related to general publication and study information (group 1)

For descriptive purposes, we will code the following information: Journal, topic of the systematic review (freetext; post-hoc grouping), (sub-)clinical outcome investigated in children or adolescents (freetext; post-hoc grouping), whether or not the systematic review assessed intervention effects (yes/no), and the eligible age group(s) of the systematic review (infancy [0–24 months], childhood [2–10 years], adolescence [11–20 years]), number of included publications (preferred over number of studies due to easier coding access to this information), number of included participants (if reported). In addition, we also code further qualifying information for some transparency items (e.g., type of estimator for variance components).

##### 2.8.1.2 Transparency-related items (group 2)

We focus on transparency-related criteria as reported within the PRISMA guidelines, given their ubiquitous use and current best-practice recommendation within health-related systematic reviews (Kolaski et al., 2023), including those from Psychology (Steil et al., 2022). To this end, we used transparency-related indicators as formalized within the core PRISMA guidelines, version 2020, within their explanation and elaboration document (Page et al., 2021b) as well as the PRISMA-S guidelines for search strategies (Rethlefsen et al., 2021), which were published within the same year and can thus be assumed to have a similar level of uptake.

While the PRISMA guidelines provide detailed guidance for review authors, they cannot be readily used for assessing transparency of existing systematic reviews, as the items are typically broad and consist of several transparency-related recommendations, which would need to be assessed (and scored) individually. To adapt the PRISMA indicators of transparent reporting for our purposes, we proceeded in the following steps: First, we manually checked codebooks of published meta-reviews within the social and medical sciences gathered within an unsystematic literature search (Page et al., 2016, 2018; Polanin et al., 2020; Chow et al., 2021; Chin et al., 2022; López-Nicolás et al., 2022; Nguyen et al., 2022; Silva et al., 2023; Wong and Bouchard, 2023; Rethlefsen et al., 2024; Sandoval-Lentisco et al., 2024) and grouped items according to their superordinate PRISMA items (e.g., eligibility criteria, information sources). Second, we cross-checked the content of these items against the “essential items” list as reported within the PRISMA explanation and elaboration document (Page et al., 2021b) and adapted them where necessary. Third, for all essential items not covered, we created new items within the research team.

We did not create new items or adapted existing items for PRISMA essential items that would require comparison with primary studies (e.g., whether assumptions were made regarding missing or unclear information within primary studies; whether all effect sizes possible were extracted) or the review protocol (e.g., whether all planned analyses were conducted), as these analyses are excessively time-consuming and typically conducted within separate meta-scientific investigations, (Tricco et al., 2016; see, e.g., Lakens et al., 2017; Maassen et al., 2020). PRISMA is geared toward systematic reviews of interventions. Thus, we also adapted items to encompass a broader spectrum of reviews where necessary (e.g., a meta-analysis investigating prevalence rates does not need to define eligibility criteria for comparators and interventions). For items related to the transparency of the search procedure, we combined items from PRISMA with the more detailed items from PRISMA-S (Rethlefsen et al., 2021). Following (Wong and Bouchard, 2023), we opted against coding abstracts of systematic reviews (as formalized within the respective PRISMA guidelines for abstracts), because we deemed length and content of abstracts as too journal-dependent for a meaningful analysis. For transparency of reporting of effect sizes, we also added an item (adapted from López-Nicolás et al., 2022) related to reporting of formulae for effect size calculation. While this is not mentioned explicitly within PRISMA guidelines, it is an important aspect of meta-analytic reproducibility (Lakens et al., 2017; Maassen et al., 2020). We also added items related to the reporting of interrater reliability of the screening and coding procedure (irrespective of its value, as would be assessed via AMSTAR 2), as well as regarding thresholds for reporting bias indication with statistical methods (Siegel et al., 2022). To better track the rising use of generative AI within the systematic review process as well as manuscript preparation (Ge et al., 2024) we also included an item on the use of generative AI within the disclosure section (note that some forms of generative AI use are also coded within the automation tools section). In all, we created (143) or adapted (25) 168 items related to transparency of meta-analytic systematic reviews (see Table 4 and Supplementary materials S3, S4).

**Table 4:** Correspondence between transparency domains, PRISMA items, and items within our transparency checklist.

Section	PRISMA item number	Number (Max. Points) or range of number of items (Max. Points) within category
Domain		
Title	1	1 (1)
Introduction	3, 4	4–6 (4–6)
Eligibility criteria	5	6 (3)
Systematic search procedure	6, 7, 8^a^, 16; all PRISMA-S items	Overall: 8–27 (8–24) Methods: 6: 1–11 (1–11), 7: 2–7 (2–7); 8: 3–6 (3–4) Results: 16: 2–3 (2–3)
Data collection	9^a^, 10, 17	Overall: 6–12 (6–11) Methods: 5–11 (4–9) Results: 1 (2)
Risk of bias	11, 18	Overall: 5–9 (5–8) Methods: 3–7 (3–6) Results: 2 (2)
Effect measures & statistical synthesis	12^b^, 13, 19, 20	Overall: 13–26 (11–22) Methods: 12: 3–4 (3–4), 13: 6–11 (4–7) Results: 19: 2 (2); 20: 2–9 (2–9)
Reporting bias assessment	14^c^, 21	Overall: 2–10 (2–8) Methods: 1–8 (1–6) Results: 1–2 (1–2)
Certainty assessment	15, 22	Overall: 7–12 (5–9) Methods: 4–10 (3–7) Results: 2 (2)
Discussion	23	4 (4)
Open science practices^d^	24, 27	2–5 (2–4)
Disclosure practices	25, 26	2–3 (2–3)
Supplementary scores		
Automation tools	8, 9, 11, 15, 16	1–10 (1–10)
CCAP	–	6–7 (6–7)

^a^Item about reporting of interrater agreement (in case of more than one rater) added.^b^Item about reference to formulae to calculate effect sizes added.^c^Item about thresholds for statistical bias detection methods added.^d^To be analyzed in detail within separate investigations (preregistration to be amended, see below). Range items (excepting supplementary score): 59–121; range maximum points (excepting supplementary score): 53–104.

As outlined within the introduction, scholars have voiced a repeated interest in and need for PRISMA (i.e., transparency) guidelines specifically for systematic reviews involving pediatric populations (i.e., the PRISMA-C guidelines; Kapadia et al., 2016). Presently (November 2024), these guidelines have not been published, but a proposition of possible adaptations exists (Farid-Kapadia et al., 2017a). We used these propositions to develop seven transparency-related items unique to systematic reviews from the field of CCAP. These involve: Mentioning the pediatric population of interest within the title (1) and study objectives (2; two separate items); providing justification for the age group(s) studied (3); providing replicable age-related eligibility criteria (4); providing a rationale for search terms related to the eligible age group (5); providing a field-specific classification of the strength of effect sizes (6); and discussing practical implications of findings for children, adolescents, and/or their caregivers (7). For descriptive purposes, we further developed items to assess whether the authors used search terms or filters related to the eligible age group(s), whether they used tested pediatric search filters (both not included in transparency scores), and whether they considered (within the eligibility criteria) or coded (within the data items) ethics-related criteria regarding child and adolescent populations within the reviewed primary studies (e.g., provision of IRB approval, waiver of parental consent).

##### 2.8.1.3 Quality-related items (group 3)

To assess methodological quality, we will use AMSTAR 2 (Shea et al., 2017), which is the currently recommended and widely used tool for assessing quality of systematic reviews within meta-scientific investigations (Kolaski et al., 2023). With AMSTAR 2 it is possible to assess the methodological quality of (meta-analytic) systematic reviews irrespective of content domain within 16 items, with seven of them being deemed as critical (Shea et al., 2017). The reliability and validity of AMSTAR 2 has been found to be acceptable (Kolaski et al., 2023). We chose to not adapt AMSTAR 2 in any way to facilitate comparisons with other meta-scientific studies interested in the methodological quality of systematic reviews. Thus, we will use AMSTAR 2 as recommended within its accompanying documentation. [MS, SF, JR] will use AMSTAR 2 to assess the methodological quality of meta-analytic systematic reviews with MS assessing all eligible reviews and SF and JR each assessing half (corresponding to their coding of transparency-related items). We will pilot our shared understanding of AMSTAR 2 based on two eligible reviews and, if necessary, code a third to reach consensus.

We deliberately did not include further CCAP-specific items related to methodological quality as we did for transparency. The reason is that there is currently, to the best of our knowledge, no broader consensus of experts within the field (or discussion thereof) what these CCAP-specific quality criteria could entail. We believe it is more conducive to the field that such a consensus is reached among different groups of experts and researchers rather than providing our idiosyncratic assessment of CCAP-related methodological quality here. Note that, for transparency, while a fully fleshed out PRISMA-C guideline has not been published, these deliberations between experts have already been taking place (Kapadia et al., 2016; Farid-Kapadia et al., 2017a, 2017b) and could be adapted here.

##### 2.8.1.4 Items generated by statcheck (group 4)

For our assessment of statistical consistency, we will use the open-source tool statcheck (Nuijten and Epskamp, 2024) in its version as an R package (CRAN release at date of analysis; currently version 1.5). Statcheck searches for results of NHSTs within manuscripts, recomputes *p*-values from reported test statistics (e.g., *t*-statistics, degrees of freedom), and compares the recomputed with the reported *p*-value (Nuijten et al., 2016; Nuijten and Epskamp, 2024). In case of a mismatch between the reported and recomputed *p*-values (with rounding and, possibly, one-sided testing being taken into account), the result is flagged as an inconsistency. If one *p*-value would be regarded as statistically significant (based on a chosen threshold, e.g., *α* = 0.05) and the other would not (e.g., the reported but not the recomputed *p*-value is significant), the result is flagged as a gross inconsistency (Nuijten and Epskamp, 2024). It is important to note that it does not necessarily need to be the *p*-value that is incorrectly reported. Also, a reporting error in test statistics or degrees of freedom (e.g., a typo) could result in a recomputed *p*-value that is different from the one that was reported (Nuijten and Epskamp, 2024).

Technically, statcheck first converts a .pdf- or .html-file to plain text, whereby .html-files are preferable due to fewer issues in plain text conversion (Nuijten et al., 2016). Second, statcheck detects and then extracts test statistics, degrees of freedom, and *p*-values based on predefined string templates in APA format that are matched against the plain text. Third, statcheck recomputes the *p*-values and, fourth, compares it with the reported *p*-value (Nuijten et al., 2016). More information regarding technical details of statcheck can be found elsewhere (Nuijten et al., 2016; Nuijten and Polanin, 2020; Nuijten and Epskamp, 2024).

Several requirements need to be met for statcheck to function properly based on its mode of working (Nuijten and Epskamp, 2024): First, the results must be reported according to APA style. Second, the result needs to be reported in full, i.e., the test statistic, the degrees of freedom (if applicable) and the *p*-value need to be reported. Third, the result must be reported within the text and not within tables due to its reliance on predefined sets of strings to be detected (Nuijten and Epskamp, 2024). A validation study (based on primary studies) comparing manual extraction of results and statcheck found that statcheck was able to extract 67.5% of reported results (Nuijten et al., 2016). Thus, we expect only a subsample of all reported test statistics to be analyzable via statcheck, namely those reported in APA format in the main text. As a comparison, only 21.6% of meta-analytic systematic reviews within the social sciences have been found to contain at least one fully APA-formatted result from a NHST (Polanin and Nuijten, 2018).

We plan to use statcheck:statcheck() (Nuijten and Epskamp, 2024) in its default settings, including: (i) extraction of all test statistics; (ii) assumption of two-tailed tests; (iii) an alpha level of 0.05; (iv) *p* ≤ *α* (vs. *p* < *α*) is considered to be significant; (v) “*p* = 0.000” (as opposed to “*p* < 0.001”) is considered to be an error; (vi) correction for one-sided tests if mentioned within text; (vii) only *p*-values that comply fully with APA-formatting are included in the output. We will also use default settings for classifications of inconsistencies (i.e., mismatch between reported and recomputed *p*-values not explained via rounding or one-sided testing) and gross inconsistencies (the reported *p*-value is < 0.05 and the recomputed is not, or vice versa).

Statcheck’s performance in detecting statistical inconsistencies (given necessary preconditions) has been assessed elsewhere (Nuijten et al., 2016, 2017) and is not the focus of this paper. To gauge the accuracy and completeness of the data extraction procedure within our study (statcheck’s main source of errors or non-detection), we will randomly draw 10% (rounded to the next integer) of eligible meta-analytic systematic reviews where statcheck was able to detect at least one NHST reported in APA-style. For example, if statcheck was able to detect at least one NHST reported in APA-style in 13 meta-analytic reviews (21.6% of the estimated 60 studies), we will randomly select two meta-analytic systematic reviews. From these, we will manually extract all reported test statistics (including from tables) and compare the number of extracted statistics to those extracted by statcheck. Reasons for failure of detection (e.g., incomplete test statistic, reported in a table) will be noted.

By default, statcheck in its current CRAN version 1.5 (Nuijten and Epskamp, 2024) provides the following output per analyzed document (here: review), excepting variables extracted to facilitate manual post-hoc checks related to the correct import: type of test statistic (e.g., *Q*), first and second degrees of freedom (if applicable), test statistic, reported *p*-value, recomputed *p*-value, errors (true/false; i.e., whether reported and recomputed *p*-values are incongruent), decision errors (true/false; i.e., whether either the reported or recomputed *p*-value is significant and the other is not), whether or not text indicated one-sided testing was detected (true/false), and the proportion of correctly APA-formatted test statistics (as an indication of possibly missed results due to reporting issues). Statcheck also alerts users to possible manual checks (e.g., checking the significance level in case a result would have been correct in case of *α* = 0.01). We will respond to these alerts by checking full-texts and adapting the statcheck input for this review if necessary (e.g., *α* = 0.01). Following (Nuijten and Polanin, 2020), we will also conduct manual checks of errors classified as decision errors.

### 2.9 Planned analyses

Our aim is to assess meta-analytic systematic reviews from seven leading journals within the field but not to generalize to a wider population (which would, ideally, include a random sample). Thus, we will generally present our results descriptively and will not, for example, provide confidence intervals (e.g., for proportions). Notable exceptions include our exploratory analyses, where we will provide *p*-values and/or confidence intervals for the sake of completeness, but emphasize the descriptive nature of our analyses. We assume a two-tailed *p*-value of < 0.05 as our threshold for significance for these purposes.

#### 2.9.1 Descriptive sample statistics

We will report descriptive sample statistics (counts and proportions for categorical variables, *M*, *SD*, range for continuous variables) for the following variables, possibly stratified by journal if deemed valuable: Journal, type and topic of the meta-analytic systematic reviews, (sub-)clinical conditions studied within pediatric populations, pediatric age groups, type of effect size metric, type of meta-analytic model (fixed-effect/equal-effects model; two-level random-effects model; three-or-more-level random-effects model), number of included publications, number of participants.

#### 2.9.2 Research question 1 (transparency)

##### 2.9.2.1 Scoring and primary metric

In general, we will assign 0 to all transparency-related items where necessary information was not reported and 1 to those where necessary information was reported. In some cases, answer options allow for scoring of a “partial” reporting of information, for which we will assign 0.5 points. Evidently, not all items are applicable for every meta-analytic systematic review. For example, for a review that did not report undertaking a risk of bias assessment, we will code all respective items related to transparent reporting of risk of bias assessment (e.g., specification of tool; reporting of number of authors involved in assessment) as “not applicable” (i.e., as a missing value). Importantly, this means that we do not code these items as a lack of transparency (i.e., by awarding 0 points). Rather, the lack of the risk of bias assessment is related to and reflected within study quality, which is assessed via AMSTAR 2 items. Notable exceptions include, for example, the reporting of studies excluded during full-text screening together with a justification, where transparent reporting is a quality criterion within AMSTAR 2. Additionally, some essential items within the PRISMA guidelines were explicitly phrased as “if-statements” (Page et al., 2021a) and thus only applicable to reviews where these conditions were met (e.g., if the systematic review assesses an intervention, it should be briefly described how this intervention works within the introduction section). In these cases, we also code items as “not applicable” if the condition is not met.

This means that every systematic review within our sample will possibly have a different maximum possible score, because different sets of items will apply to different reviews. We solve this through calculating the percentage of maximum possible (POMP) score (Cohen et al., 1999) as our primary metric, where the transparency score is expressed as the tallied score for a given review divided by the maximum possible score for this review. Thus, as 0 is the minimum score for all reviews, the POMP score of review *i* can be expressed as: POMP_i_ = (Score_i_/Max.Score_i_) * 100. Table 4 reports the possible range of items and points for all broad transparency-related domains.

##### 2.9.2.2 Scores

We will form several scores based on our coding of transparency-related items as well as based on different weighting schemes for PRISMA domains. As outlined above, all scores will be expressed as POMP (see above).

First, we will calculate overall transparency scores by forming POMP scores based on all transparency items excepting those related to automation tools and CCAP (which will be scored separately, see below). We will form two different version of these overall scores: First, we form an unweighted version where it is not considered that some PRISMA items comprise more individual essential items. For example, the PRISMA item related to a review’s title (item #1) comprises one essential item and is thus awarded only one point, whereas item #12 (effect measures) comprises three essential items (and we added a fourth related to the provision of formulae for calculation of effect sizes, see above) and is thus awarded four points. Second, we will thus also calculate a weighted version, where each PRISMA item is weighted equally (i.e., contributing equally to the overall score, regardless of the number of essential items). For our example above, this would mean that the essential item for PRISMA item #1 would still be scored with one point, but our essential items for PRISMA item #12 would be awarded 1/4 = 0.25 points each.

Second, this broad score will be supplemented by a finer-grained, domain-based POMP score to elucidate transparent reporting practices within different domains of a review. These domains include the following sections (see Table 4 for correspondence between these domains, the PRISMA items, as well as our items based on PRISMA essential items within the broad items): Title, introduction, eligibility criteria, systematic search procedure, data collection, risk of bias, statistical synthesis (i.e., meta-analysis), reporting bias assessment, certainty assessment, discussion, open science practices, disclosure practices. Domains will be scored as missing in case all transparency-related items were coded as “not applicable” for this domain (e.g., a review that did not conduct a certainty assessment will not be scored within this domain).

Third, we will compute a separate automation tool score for those reviews that used automation tools within sections systematic search procedure (PRISMA item #8), data collection (#9), and risk of bias (#11), as outlined within the PRISMA guidelines (Page et al., 2021a). As the uptake of automation tools within research synthesis is an on-going process (Van Altena et al., 2019), we deemed it reasonable to score and report usage of these tools separately from reporting of other, more established, components of a review.

Fourth, we will compute a separate, CCAP-related transparency score that focuses on transparent reporting as it relates to CCAP-specific information. Because we derived seven CCAP-related transparency items, we will report adherence to transparent reporting for every item separately and will also form an overall score.

##### 2.9.2.3 Presentation of results and answer to RQ 1

We will report results per item within Supplementary material (overall and stratified by journal). Within the main text, we will present *M* and *SD* of POMP scores (overall; by domain; automation tools; CCAP) across all reviews in two version: First, an unweighted version (i.e., mean score across all reviews) and second, a weighted version where differing numbers of reviews across journals are considered. For this weighted version, we will form a grand mean of the mean scores by journal. For the overall score, this will be crossed with the weighted and unweighted scoring related to PRISMA items (see above), resulting in 2 (PRISMA) × 2 (journal) = 4 scores. Results by journal will similarly be made available within Supplementary material. Within the main text, we will focus on emphasizing broad patterns found within the results and highlight certain domains (e.g., particularly transparent or non-transparent parts of reviews) as deemed insightful. Findings will be presented in tabular form, narratively, and through visualization (e.g., stacked bar plots). To answer RQ 1, we will focus on overall and domain-based scores, which we will contextualize within the discussion both with regard to between-journal variability as well as with regard to findings from other (sub-)disciplines. Our aim is not to provide readers with a single percentage score, but rather to elucidate areas of high and low transparency as well as how the latter could be improved.

#### 2.9.3 Research question 2 (quality)

##### 2.9.3.1 Scoring

We will score AMSTAR 2 based on its accompanying information. For our overall rating of the confidence in a review, we will adhere to recommendations as given within the original AMSTAR 2 publication, where high confidence is assigned to reviews with none or only one non-critical weakness (i.e., in a domain deemed as non-critical); moderate confidence where more than one non-critical weakness has been identified, low in cases where at least one flaw in a critical domain has been identified, and critically low when more than one critical flaw has been identified (Shea et al., 2017).

AMSTAR 2 does not provide an overall score to avoid concealing critical flaws within a review (Shea et al., 2017). We agree with this notion and will thus focus on presenting results for single items (see below) as well as an overall assessment of confidence within the review. However, for our exploratory analyses (see below), we will need to assign numeric values to AMSTAR 2 ratings. For the purpose of these analyses, we will assign “Yes” ratings with a score of 1, “Partial Yes” ratings with a score of 0.5, and “No” ratings with a score of 0 and create a sum score of all items. If, similar to our transparency ratings, we encounter instances where an AMSTAR 2 item is not codable for a given review, we will revert to POMP scores if necessary.

To answer RQ 2, we will focus on results from single items as well as overall confidence ratings. As for RQ 1, both will be contextualized within the discussion section.

##### 2.9.3.2 Presentation of results

We will present results per item (items deemed as critical within AMSTAR 2, i.e., #2, #4, #7, #9, #11, #13, #15, will be highlighted) as well as for the overall confidence rating as recommended (Shea et al., 2017). Similar to results from our transparency assessment, we will report unweighted and weighted results that do not (unweighted) and do (weighted) take differing numbers of reviews within journals into account. We will present results in tabular form, narratively, and visually (e.g., by using stacked bar plots) and within main text and Supplementary material based on the nature of the results.

#### 2.9.4 Research question 3 (consistency)

For RQ 3, we will report the number of errors (i.e., inconsistencies between reported and re-computed *p*-values) as well as the number of gross decision errors (i.e., the assessment of significance differs based on reported and re-computed *p*-values). Both will be reported overall (unweighted and weighted) as well as stratified by journal. To answer RQ 3, we will focus on these two types of errors and contextualize them within the discussion section in comparison with other statcheck-based evaluations of corpora of primary studies and meta-analytic systematic reviews.

#### 2.9.5 Exploratory analyses and effect strength interpretation

We will conduct a series of exploratory analyses, all of which are expressed as associations between two variables (Pearson *r* or Cohen *d*); the latter calculated according to default settings within effectsize:cohens_d() (Ben-Shachar et al., 2020), corresponding to formulae 4.2–4.6 as reported within Borenstein et al. (2021), where nesting within journal is not taken into account due to the descriptive nature of our analysis. Specifically, we will test: (1) Associations between reviews’ transparency and quality, as expressed by transparency and quality overall scores; (2) associations between reviews’ transparency and inconsistent reporting, as expressed by overall transparency scores and dummy-coded variables related to number of errors (i.e., at least one error within the review) and number of decision errors (i.e., at least one decision error within the review); (3) associations between reviews’ transparency scores and the journal’s TOP factor; (4) associations between reviews’ transparency and self-reported adherence to PRISMA guidelines (yes vs. no), (5) associations between a reviews’ transparency and journal page limit (no page limit vs. long page limit; no page limit vs. short page limit; see Table 3). Transparency scores will always be used twice for analysis (weighted/unweighted by PRISMA criterion).

Regarding effect size interpretation, there are currently no established guidelines for what constitutes meaningful effect strength within meta-reviews such as this. Thus, we resort to an *ad hoc* approach based on previous meta-scientific findings within the literature and our own considerations. For effects expressed as Cohen *d* (analyses 2, 4, 5), we assume a difference of 5% of transparency scores between groups as practically meaningful. Based on findings within Psychology (Polanin et al., 2020), which align with standard deviations of PRISMA scores found in other fields (Dhillon et al., 2022; Peña et al., 2022; Streck et al., 2022), we assume a pooled standard deviation of 14%, which would result in a *d* = 5/14 = 0.35, which we round down to 0.30 to be in line with well-established guidelines for typical, lower thresholds of small-to-medium-sized effects (Cohen, 1988). For effects expressed as Pearson *r* (analyses 1, 3), we turn to previous associations found between PRISMA and AMSTAR 2 scores (to our knowledge, PRISMA scores have not been correlated with journals’ TOP scores) that have been documented in the literature, (e.g., Rainkie et al., 2020; Peña et al., 2022; Javidan et al., 2023; Sehmbi et al., 2023), ranging between 0.33 (Rainkie et al., 2020) and 0.78 (Sehmbi et al., 2023). Here, we use the lower limit of *r* = 0.30 as our assumed threshold for a meaningful effect.

### 2.10 Data management and availability plan

See Supplementary material S5.

### 2.11 Status and timeline

Upon submission to the journal and concurrent preregistration as a preprint (November, 2024), the status of our project was the following: Preliminary searches as well as piloting of the eligibility criteria and the screening procedure in a randomly drawn sample of 55 records had been conducted. The coding procedure had been piloted on two meta-analytic systematic reviews. We had not started the main search, screening, and coding procedures.

Upon reception of reviews (June, 2025), the status of the project is as follows: We have completed the systematic search and title-and-abstract screening and established a final sample of eligible meta-analytic systematic reviews. Coding for all three main pillars of this meta-review (transparency, quality, and statistical consistency) is currently underway. Reviewers’ comments did not address any steps completed in the meantime that would need to be repeated.

### 2.12 Potential follow-up investigations

We plan to conduct further investigations based on the review sample obtained from this study. First, we plan to analyze adherence to open science practices of eligible meta-analytic systematic reviews in more detail within a separate report, including practical recommendations to improve adherence to open science principles within meta-analytic systematic reviews. Second, we plan to assess the computational reproducibility of meta-analyses within our sample, similar to López-Nicolás et al. (2024). Third, we plan to compare review protocols and preregistrations with published reviews, similar to Sandoval-Lentisco et al. (2025). All of these investigations will be preregistered.

## 3 Discussion

This study protocol for a meta-review outlines how we plan to assess transparency, methodological quality, and statistical consistency of recent (2022–2024) meta-analytic systematic reviews published in seven leading CCAP journals. Our goal is to identify strengths in these areas and highlight opportunities for improvement. In doing so, we ultimately hope to provide further guidance for applied meta-analysts, reviewers, and editors within the field. To our knowledge, this is the first meta-scientific investigation focused specifically on meta-analytic systematic reviews within CCAP (see Sifers, 2002; Raad et al., 2008, for slightly dated investigations at the primary study level) and one of the first within Clinical Psychology (López-Nicolás et al., 2022). As such, it will contribute to a better understanding of the trustworthiness of meta-analytic systematic reviews within these fields (and whether and how these could be improved) and provide insights into how they compare to other subdisciplines of Psychology in terms of reporting transparency, quality, and statistical consistency.

Our transparency assessment tool is based on previous literature (Page et al., 2016, 2018; Polanin et al., 2020; Chow et al., 2021; Chin et al., 2022; López-Nicolás et al., 2022; Nguyen et al., 2022; Silva et al., 2023; Wong and Bouchard, 2023; Rethlefsen et al., 2024; Sandoval-Lentisco et al., 2024) and aligns with PRISMA essential items (vs. the broader PRISMA checklist), which allows for a nuanced, comprehensive, systematic, and field-independent assessment. We also include field-specific transparency items for CCAP, which are literature-based (Kapadia et al., 2016; Farid-Kapadia et al., 2017a, 2017b). Methodological quality will be evaluated by using AMSTAR 2 (Shea et al., 2017), enabling comparisons with previous studies. By applying statcheck (Nuijten and Epskamp, 2024), we also provide new evidence on the prevalence of reporting errors within research syntheses, adding to a predominantly primary study-based evidence base. Finally, we will assess data and code sharing practices as part of our transparency assessment, disconcertingly uncommon within other (sub-)disciplines (Polanin et al., 2020; López-Nicolás et al., 2022; Nguyen et al., 2022). This will help to shed light on open science practices within CCAP and Clinical Psychology more broadly, where uptake of these practices has been found to be low (Howard et al., 2024).

Several limitations of this planned meta-review are foreseeable at this stage. First, our sample will consist of recent meta-analytic systematic reviews from leading, high-impact CCAP journals, not a random (and thus representative) selection. Therefore, our results will likely represent an upper threshold of transparency, quality, and statistical reporting consistency and generalizations to the field cannot be drawn. For this reason, results will also be presented descriptively. We made this sampling choice for several reasons: For one, this is the first, notably unfunded, meta-scientific investigation of this kind within the field of CCAP, where an assessment of this “upper threshold” of reporting and quality characteristics might be particularly insightful for future applied and meta-scientific works in this area. Applied meta-analysts might turn to meta-analytic systematic reviews published within these outlets to guide their own work and thus domains of reporting non-transparency or lack of quality could be further perpetuated, because examples from leading journals would indicate that a review is nonetheless publishable. Similarly, reviewers and editors, tasked with assessing potentially highly impactful reviews for the field, can focus their attention on domains where reporting has been found to be lacking quality or transparency. In addition, as there is no dedicated category for CCAP (as, for example, compared to Clinical Psychology as a whole) within large-scaled scientific databases (e.g., *Web of Science, Scopus*), it is difficult to establish the sampling frame for such a randomly drawn sample. As for meta-reviews of somewhat narrower focus, the best option would be a keyword search, but it is currently unclear on how a search string encompassing all outcome domains eligible for our (or similar) investigations could look like. Thus, we opted for a journal-based strategy, as is also common within meta-scientific investigations (e.g., Polanin et al., 2020; Siegel et al., 2022). However, the sample from this meta-review might help to establish such a search string in the future, possibly enabling a more comprehensive assessment of reporting practices within CCAP.

Second, our comprehensive assessment of reporting transparency is not a published and validated tool, even though it is grounded in previous literature and aligns with current PRISMA (Page et al., 2021a) and PRISMA-S (Rethlefsen et al., 2021) guidelines. However, such a tool does (to our knowledge) not exist so far. Similarly, our CCAP-specific items are based on preparatory works for PRISMA-C guidelines, but ultimately reflect our subjective judgments about transparency-related issues relevant for the field of CCAP. Ideally, consensus among experts would guide these judgments, which exceeds the scope of this study. Thus, we view the inclusion of these items as exploratory and possibly informative for future, consensus-based work in this regard.

Third, we do not assess reporting practices requiring access to primary studies or study protocols (if existent), such as the correct extraction and computation of effect sizes, comparisons of primary outcomes within protocols and final publications, or meta-analytic reproducibility. These assessments have been found to be excessively time-consuming (Tricco et al., 2016; Lakens et al., 2017; Maassen et al., 2020) and typically warrant their own investigation. Our sample could serve as the basis for focused investigations into these domains.

Fourth, while the use of NHSTs within meta-analyses is common (averaging between 28 and 60 per publication; Polanin and Pigott, 2015; Nuijten and Polanin, 2020), the number of NHSTs reported in sufficient detail for re-analysis via statcheck is much lower (Md = 4; Polanin and Nuijten, 2018). This limitation is common to all investigations into statistical reporting consistency using statcheck (the only dedicated tool to date) and might indicate a broader problem of insufficient or unsystematic reporting of *p*-values and accompanying test statistics within meta-analyses. Still, our conclusions to be drawn from our statcheck-based analysis might be limited, also considering that statcheck is not able to identify all NHSTs provided within a paper, for example those reported within tables, and relies on results to be reported in APA style (Nuijten and Epskamp, 2024).

Fifth, we want to acknowledge that our investigation of quality focuses on methodological quality as assessed within AMSTAR 2. Thus, we do not assess aspects of quality that would require content-knowledge (e.g., the suitability of eligibility criteria or the search string) because this is not possible for such a diverse set of systematic reviews.

In conclusion, our planned meta-review holds the potential to shed light on transparency, quality, and statistical consistency of meta-analytic systematic reviews within the field of CCAP. In doing so, we hope to provide guidance for researchers, reviewers, and editors within this field and lay the foundation for future, larger-scaled works in this field.
