# Supplementary material for: Transparency, quality, and statistical consistency of meta-analytic systematic reviews in clinical child and adolescent psychology (2022–2024): study protocol for a meta-review
Source: Front Psychol. 2025 Jul 28;16:1535606. doi: 10.3389/fpsyg.2025.1535606 (PMC12336221; doi:10.3389/fpsyg.2025.1535606)
Supplement: Supplementary file 1 [file Supplementary_file_1.docx]

**PRISMA-P checklist for:**

Siegel, M., Fanninger, S., Riedel, J., & Zemp, M. (2024). *Transparency, Quality, and Statistical Consistency of Meta-Analytic Systematic Reviews in Clinical Child and Adolescent Psychology (2022-2024): Study Protocol for a Meta-Review*. <https://doi.org/10.17605/OSF.IO/QHRAU>

**NOTE:** This PRISMA-P checklist was requested as part of the submission of this protocol to a peer-reviewed journal. Because this study protocol is concerned with a meta-review (where reporting guidelines not yet exist) not all PRISMA-P items are applicable, as these would apply to systematic reviews synthesizing results from primary studies. We note these items together with a justification below.

Note that line numbers refer to this version of the preprint for transparency, where all information regarding funding disclosure, postal address etc. is provided within the manuscript (as opposed to the journal’s submission system, where separate fields are provided for this information): <https://osf.io/ah5m4>

**PRISMA-P (Preferred Reporting Items for Systematic review and Meta-Analysis Protocols) 2015 checklist: recommended items to address in a systematic review protocol***

| Section and topic | Item No | Checklist item | Line number/location (version: <https://osf.io/ah5m4>) or justification of omission |
| --- | --- | --- | --- |
| ADMINISTRATIVE INFORMATION | | |  |
| Title: |  |  |  |
| Identification | 1a | Identify the report as a protocol of a systematic review | Title (we identified it as a meta-review, a methodological overview of systematic reviews) |
| Update | 1b | If the protocol is for an update of a previous systematic review, identify as such | N/A |
| Registration | 2 | If registered, provide the name of the registry (such as PROSPERO) and registration number | Preregistered as preprint on OSF: <https://osf.io/ah5m4> |
| Authors: |  |  |  |
| Contact | 3a | Provide name, institutional affiliation, e-mail address of all protocol authors; provide physical mailing address of corresponding author | Title page; e-mail addresses of all other authors: [selina.fanninger@univie.ac.at](mailto:selina.fanninger@univie.ac.at); [juliariedel2@googlemail.com](mailto:juliariedel2@googlemail.com); [martina.zemp@univie.ac.at](mailto:martina.zemp@univie.ac.at) |
| Contributions | 3b | Describe contributions of protocol authors and identify the guarantor of the review | Title page (guarantor = project administration = MS) |
| Amendments | 4 | If the protocol represents an amendment of a previously completed or published protocol, identify as such and list changes; otherwise, state plan for documenting important protocol amendments | N/A |
| Support: |  |  |  |
| Sources | 5a | Indicate sources of financial or other support for the review | Title page (no funding) |
| Sponsor | 5b | Provide name for the review funder and/or sponsor | N/A |
| Role of sponsor or funder | 5c | Describe roles of funder(s), sponsor(s), and/or institution(s), if any, in developing the protocol | N/A |
| INTRODUCTION | | |  |
| Rationale | 6 | Describe the rationale for the review in the context of what is already known | Line numbers: 1-155 |
| Objectives | 7 | Provide an explicit statement of the question(s) the review will address with reference to participants, interventions, comparators, and outcomes (PICO) | Line numbers: 166-185 (note that the PICO framework is not applicable to the research question, but note our framing of the eligibility criteria within this framework) |
| METHODS | | |  |
| Eligibility criteria | 8 | Specify the study characteristics (such as PICO, study design, setting, time frame) and report characteristics (such as years considered, language, publication status) to be used as criteria for eligibility for the review | Line numbers: 247-415 |
| Information sources | 9 | Describe all intended information sources (such as electronic databases, contact with study authors, trial registers or other grey literature sources) with planned dates of coverage | Line numbers: 416-485 |
| Search strategy | 10 | Present draft of search strategy to be used for at least one electronic database, including planned limits, such that it could be repeated | Line numbers: 486-512 |
| Study records: |  |  |  |
| Data management | 11a | Describe the mechanism(s) that will be used to manage records and data throughout the review | Line numbers: 897-929 |
| Selection process | 11b | State the process that will be used for selecting studies (such as two independent reviewers) through each phase of the review (that is, screening, eligibility and inclusion in meta-analysis) | Line numbers: 539-572 |
| Data collection process | 11c | Describe planned method of extracting data from reports (such as piloting forms, done independently, in duplicate), any processes for obtaining and confirming data from investigators | Line numbers: 573-602 |
| Data items | 12 | List and define all variables for which data will be sought (such as PICO items, funding sources), any pre-planned data assumptions and simplifications | Line numbers: 603-736 |
| Outcomes and prioritization | 13 | List and define all outcomes for which data will be sought, including prioritization of main and additional outcomes, with rationale | Line numbers: 737-867 (we define outcomes as the transparency and quality scores as well as the errors and decision errors as identified by statcheck) |
| Risk of bias in individual studies | 14 | Describe anticipated methods for assessing risk of bias of individual studies, including whether this will be done at the outcome or study level, or both; state how this information will be used in data synthesis | Line numbers: 834-859 (description of AMSTAR 2 assessment, which is, within this meta-review, one of the outcomes) |
| Data synthesis | 15a | Describe criteria under which study data will be quantitatively synthesised | N/A (this refers to whether or not a meta-analysis is possible, which we do not plan to do) |
|  | 15b | If data are appropriate for quantitative synthesis, describe planned summary measures, methods of handling data and methods of combining data from studies, including any planned exploration of consistency (such as I^2^, Kendall’s τ) | N/A (this refers to whether or not a meta-analysis is possible, which we do not plan to do) |
|  | 15c | Describe any proposed additional analyses (such as sensitivity or subgroup analyses, meta-regression) | N/A (this refers to whether or not a meta-analysis is possible, which we do not plan to do) |
|  | 15d | If quantitative synthesis is not appropriate, describe the type of summary planned | All of our analyses are described within line numbers 737-896. |
| Meta-bias(es) | 16 | Specify any planned assessment of meta-bias(es) (such as publication bias across studies, selective reporting within studies) | N/A (our unit of analysis is a systematic review) |
| Confidence in cumulative evidence | 17 | Describe how the strength of the body of evidence will be assessed (such as GRADE) | N/A ((our unit of analysis is a systematic review) |

*** It is strongly recommended that this checklist be read in conjunction with the PRISMA-P Explanation and Elaboration (cite when available) for important clarification on the items. Amendments to a review protocol should be tracked and dated. The copyright for PRISMA-P (including checklist) is held by the PRISMA-P Group and is distributed under a Creative Commons Attribution Licence 4.0.**

*From: Shamseer L, Moher D, Clarke M, Ghersi D, Liberati A, Petticrew M, Shekelle P, Stewart L, PRISMA-P Group. Preferred reporting items for systematic review and meta-analysis protocols (PRISMA-P) 2015: elaboration and explanation. BMJ. 2015 Jan 2;349(jan02 1):g7647.*
