# Supplementary material for: Transparency, quality, and statistical consistency of meta-analytic systematic reviews in clinical child and adolescent psychology (2022–2024): study protocol for a meta-review
Source: Front Psychol. 2025 Jul 28;16:1535606. doi: 10.3389/fpsyg.2025.1535606 (PMC12336221; doi:10.3389/fpsyg.2025.1535606)
Supplement: Supplementary file 2 [file Supplementary_file_2.docx]

**Supplement S2: Journal Selection (Information Sources)**

We focus on meta-analytic systematic reviews from leading journals (based on content and citation metrics criteria) within the field of Clinical Child and Adolescent Psychology (CCAP) to examine reporting practices of the (potentially) most impactful publications within this field. This may represent an upper threshold of the uptake of good reporting practices, but as we outline in our introduction, substantial reporting non-transparency has also been identified within top-ranking journals in Psychological Science, e.g., (Polanin et al., 2020). This is the first (notably unfunded) investigation into transparency, quality, and consistency of reporting within meta-analytic systematic reviews within CCAP. Thus, we deem this approach reasonable to gain a first impression of the field and deduce practical recommendations for researchers, as well as to lay the foundation for future works within this field (e.g., meta-reviews assessing time trends or prevalence estimates on reporting practices within the field). Since there is not one single, non-invitational flagship journal dedicated to publishing systematic reviews within CCAP that we could sample from (a commonly used approach within other psychological disciplines, e.g., (Polanin et al., 2020; Siegel et al., 2022)), and we deemed a keyword search as too broad (and therefore impractical, if not impossible) to identify eligible systematic reviews, we identified eligible journals through a three-step procedure:
 First, we generated an expert-based list of journals that frequently publish CCAP-related meta-analytic systematic reviews (see <https://osf.io/4v3zr>, for journal names). This initial list was created by the senior author (MZ), a professor of Clinical Child and Adolescent Psychology with strong expertise and knowledge of the field, as well as the first author (MS), a PhD student within the field of CCAP with extensive expertise in conducting research syntheses. This list was then supplemented by journals examined within previous (now slightly dated) meta-scientific examinations of primary studies within this field (Raad et al., 2008; Sifers, 2002). Due to the large disciplinary overlap of Clinical Psychology and Psychiatry (see main text: ‘Conceptual Scope Regarding the Field of Clinical Child and Adolescent Psychology’), we also included journals with a focus on Child and Adolescent Psychiatry. Note, however, that meta-analytic systematic reviews published within these journals must still comply with our eligibility criteria relating to CCAP, as defined above. General (i.e., adult-focused) journals from the field of Psychiatry as well as multidisciplinary journals covering child and adolescent health more broadly (e.g., *Lancet Child & Adolescent Health*, *Journal of Adolescent Health*) or pediatric journals (e.g., *Pediatrics*, *JAMA Pediatrics*) were not considered.

Second, we retrieved the disciplinary categories as indexed within *Web of Science* (Social Science Citation Index [SSCI]) for these journals and identified those with potentially further eligible journals within the field of CCAP, i.e., *Psychology, Clinical* and *Psychology, Developmental*. We deliberately did not cross-reference journals that were indexed by both categories (e.g., *Psychology, Clinical* and *Psychology, Developmental*), as our search revealed that impactful journals within the field were not always indexed at this intersection (e.g., *Child Development* frequently publishes meta-analytic systematic reviews with a clinical focus but is not indexed as *Psychology, Clinical*). We briefly considered the category *Psychology, Educational* but a preliminary search of top-ranking journals within this category (e.g., *Educational Psychology Review*) revealed that these journals’ dominant focus is on non-clinical issues. We ranked journals within the selected categories in descending order by their 2022 impact factor (as provided by Clarivate’s Journal Citation Reports) and again checked for eligibility based on content-related criteria (i.e., frequent publication of CCAP meta-analytic systematic reviews) and expert opinion. We did not consider (a) disorder-specific journals (e.g., *Journal of Anxiety Disorders*) to avoid overreliance on a specific subfield within CCAP, journals with a strong focus on health psychology (e.g., *Health Psychology Review*), or (c) invitation-only journals (e.g., *Annual Review of Clinical Psychology, Clinical Child and Family Psychology Review*). New journals retrieved from this step were added to our list.

Third, we checked through a somewhat more systematic procedure whether the journals on this list frequently published meta-analytic systematic reviews by tallying (a) the overall number of articles published between 2022 and 2023 (as indexed in *Web of Science, Core Collection*; May 2024), (b) the number of these articles that contained the term “meta-analy*” in the publication title, and (c) the number of these articles that contained the terms “meta-analy*” and “child*” in the title (relevant for multidisciplinary and clinical journals). As preliminary criteria, we considered journals that published five or more articles that included the terms “meta-analy*” and “child” in the title (absolute count; note that this is a low threshold because we did not search for adolesc*), and where the share of these publications of all meta-analyses published within 2022-2023 exceeded 10% (relative count). Ultimately, this yielded a list of seven journals we considered to be representative of the top-ranking journals publishing meta-analytic systematic reviews within the field of CCAP (in alphabetical order; see <https://osf.io/4v3zr> for the full list generated through our three-step procedure): *Child Development*, *Clinical Psychology Review*, *Development and Psychopathology*, *European Child & Adolescent Psychiatry*, *Journal of Child Psychology and Psychiatry*, *Journal of the American Academy of Child and Adolescent Psychiatry*, and *Psychological Bulletin*. All journals are indexed in *Web of Science* (*Core Collection*, as subscribed by the University of Vienna). Thus, we rely on this database as our single information source.

**References**

Polanin, J. R., Hennessy, E. A., & Tsuji, S. (2020). Transparency and reproducibility of meta-analyses in Psychology: A meta-review. *Perspectives on Psychological Science*, *15*(4), 1026–1041. https://doi.org/10.1177/1745691620906416

Raad, J. M., Bellinger, S., McCormick, E., Roberts, M. C., & Steele, R. G. (2008). Brief report: Reporting practices of methodological information in four journals of pediatric and child psychology. *Journal of Pediatric Psychology*, *33*(7), 688–693. https://doi.org/10.1093/jpepsy/jsm130

Siegel, M., Eder, J. S. N., Wicherts, J. M., & Pietschnig, J. (2022). Times are changing, bias isn’t: A meta-meta-analysis on publication bias detection practices, prevalence rates, and predictors in industrial/organizational psychology. *Journal of Applied Psychology*, *107*(11), 2013–2039. https://doi.org/10.1037/apl0000991

Sifers, S. K. (2002). Reporting of demographics, methodology, and ethical procedures in journals in Pediatric and Child Psychology. *Journal of Pediatric Psychology*, *27*(1), 19–25. https://doi.org/10.1093/jpepsy/27.1.19
