# Supplementary material for: Transparency, quality, and statistical consistency of meta-analytic systematic reviews in clinical child and adolescent psychology (2022–2024): study protocol for a meta-review
Source: Front Psychol. 2025 Jul 28;16:1535606. doi: 10.3389/fpsyg.2025.1535606 (PMC12336221; doi:10.3389/fpsyg.2025.1535606)
Supplement: Supplementary file 3 [file Supplementary_file_3.pdf]

Supplement 3 for

Siegel, M., Fanninger, S., Riedel, J., & Zemp, M. (2024). Transparency, Quality, and Statistical Consistency of Meta-Analytic Systematic Reviews in Clinical Child and Adolescent Psychology (2022-2024): Study Protocol for a Meta-Review. https://doi.org/10.17605/OSF.IO/QHRAU

Correspondence: magdalena.siegel@univie.ac.at

Version 1.0, 2024-11-02

Table of Contents

General Items 4
Descriptive information about the review 4
Adherence to Reporting Guidelines 6
CCAP-specific items 7
Descriptive Items 7
Transparency Items 8
Transparency Items (Including Filter and Qualification Items) 10
Title 10
Introduction 10
Eligibility Criteria 13
Systematic Search Procedure (Methods) 16
Systematic Search Procedure (Results) 28
Data Collection (Methods) 29
Data Collection (Results) 35
Risk of Bias (Methods) 35
Risk of Bias (Results) 38
Effect Measures and Statistical Synthesis (Methods) 39
Effect Measures and Statistical Synthesis (Results) 48
Reporting Bias Assessment (Methods) 51
Reporting Bias Assessment (Results) 55
Certainty Assessment (Methods) 56
Certainty Assessment (Results) 59

Discussion 59
Automation Tools 60
Open Science Practices 65
Disclosure Practices 69

Items

General Items

General items are coded for descriptive purposes only.
For items included in transparency scores, bolded text indicates which answers are awarded points, whereby the numbers in parentheses indicate the points awarded. In more complex scoring cases (e.g., several individual items pertain to one essential item), the scoring is noted at the bottom of the respective cell.
Domains listed in column "Transparency Domain" correspond to those listed in Table 4 within the main manuscript. Empty cells in this column indicate that the respective item does not count towards the transparency score.
Filter items indicate that following transparency items will only be coded for those with a respective answer (and coded as not applicable otherwise).
Qualification items qualify some transparency items in more detail (and do not count towards the transparency score). For example, a transparency-related item would be whether the estimator for variance components (e.g., τ^2) is reported, whereas the corresponding qualification item provides more detail on the type of estimator (e.g., ML) for descriptive purposes only.

Descriptive information about the review

ID	Text	Source
g01	Publication ID (Freetext) as in screening sheet.	New item
g02	Journal<br>a) Child Development<br>b) Clinical Psychology Review<br>c) Development and Psychopathology<br>d) European Child & Adolescent Psychiatry<br>e) Journal of Child Psychology and Psychiatry<br>f) Journal of the American Academy of Child and Adolescent Psychiatry	New item

	g) Psychological Bulletin	
g03	Topic of systematic review (Freetext). *E.g., "Associations between overparenting and child depression, anxiety, and internalizing symptoms" (Zhang & Ji, 2023).*	New item
g04	(Sub-)clinical condition examined (in children) (Freetext). *E.g., Internalizing symptoms; depressive symptoms*	New item
g05	Assessment of intervention: Does the systematic review include a synthesis of intervention effects?<br>*Note that the primary studies do not need to be RCTs; synthesis of pre-post effect sizes is fine as well.*<br>a) Yes<br>b) No	New item
g06	What is the age group eligible for the systematic review? (Select all that apply)<br>*Code based on eligibility criteria*<br>a) Infants (0-24 months)<br>b) Children (2-10 years)<br>c) Adolescents (11-20 years)<br>d) Unclear	New item
g07	What is the age group eligible for the systematic review? (Select all that apply)<br>*Code based on mean sample age of primary studies (fallback option)*<br>a) Infants (0-24 months)<br>b) Children (2-10 years)<br>c) Adolescents (11-20 years)<br>d) Unclear	New item
g08	What is the date the article was first submitted to the journal?<br>*Use YYYY-MM-DD format.* (Freetext)	New item
g09	What is the number of publications included in the review? (Freetext)	New item

	Only code if explicitly mentioned (no need to count).	
g10	What is the number of children/adolescent participants included/reported on in the review?<br>*Only code if explicitly mentioned. Also code if parents reported on (index)children.*	New item
g11	What is the number of parent participants included in the review?<br>*Only code if explicitly mentioned. Code NA if no data on parents was collected.*	New item

Adherence to Reporting Guidelines

**ID**	**Text**	**Source**	**Category**
r01	Did the author(s) state the use of any reporting guidelines? It is important to note that this item is referred to guidelines for correct reporting (e.g., MARS), not the use of guidelines for how to do a meta-analysis (e.g., Cochrane).<br>a) Yes, PRISMA<br>b) Yes, other guideline (Freetext)<br>c) No	Adapted from Lopez-Nicolas et al., 2022	Adherence to Reporting Guidelines
r02	If the author(s) reported using the PRISMA guidelines: Did the author(s) present a PRISMA flow chart for the study flow?<br>a) **Yes**<br>b) No	New item	Adherence to Reporting Guidelines

CCAP-specific items

Descriptive Items

ID	Text	Source
c05	Did the author(s) use search terms or filters specific to the eligible age group?<br>a) Yes<br>b) No<br>c) Unclear	New item
c07	Did the author(s) use tested pediatric search filters?<br>a) Yes<br>b) No	New item
c10	Did the author(s) consider (eligibility criteria) or code (data items) ethics-related criteria with regard to child/adolescent populations (e.g., IRB approval, waiver of parental consent)?<br>a) Yes – eligibility criteria<br>b) Yes – data items<br>c) Yes – both<br>d) No	New item

Supplement 3- Codebook

Transparency Items

ID	Text	Source	Category	Transparency Domain
c01	Does the title include the systematic review's focus on children and/or adolescents?<br>a) **Yes (1)**<br>b) No	New item	CCAP	CCAP
c02	Did the author(s) reference their target population within their research objectives/questions/hypotheses?<br>*Target population as referenced by authors within title/abstract needs to be mentioned within O/Q/H or within the respective paragraph, where it is clear that the referenced target populations refer to the following O/Q/H.*<br>a) **Yes (1)**<br>b) No	New item	CCAP	CCAP
c03	Did the authors justify the eligible age group?<br>*Code as yes if justified either within the introduction or the eligibility criteria*<br>a) **Yes (1)**<br>b) No	New item	CCAP	CCAP
c04	Did the author(s) provide replicable age-related eligibility criteria?<br>*Code as yes if the eligibility criteria are sufficient enough to replicate the eligibility assessment within primary studies. This would need to include information about the sample age within primary studies (e.g., mean, range). Statements*	New item	CCAP	CCAP

	such as "the systematic review includes studies assessing children from 5-9" are insufficient, because it is unclear whether this refers to primary studies' mean or range.<br>a) **Yes (1)**<br>b) No			
c06	If the author(s) used search terms or filters specific to the eligible age group:<br><br>Did the author(s) provide a rationale for these search terms? *Check if search terms correspond broadly to eligibility criteria*<br>a) **Yes (1)**<br>b) No	New item, if	CCAP	CCAP
c09	Did the author(s) state thresholds/ranges used to interpret the size of the effect(s) and the rationale by drawing on CCAP-specific sources?<br>*A citation sufficiently covers the threshold/range and rationale. Code not applicable for prevalence studies*<br>a) **Yes (1)**<br>b) No	New item	CCAP	CCAP
c08	Did the author(s) discuss implications of their findings particularly for children, adolescents, and caregivers?<br>a) **Yes (1)**<br>b) No	New item	CCAP	CCAP

Transparency Items (Including Filter and Qualification Items)

Title

Note that the second essential item ("report an informative title…") was skipped in favor of the more informative item c01 (CCAP).

ID	PRISMA Item	Text	Source	Category	Transparency Domain
01.01	Title	Which of the following terms are in the title of the review?<br>*Select only one.*<br>a) **Systematic Review (1)**<br>b) Meta-Analysis (or Meta-Regression)<br>c) **Both Systematic Review and Meta-Analysis (1)**<br>d) Another term indicating the conduction of a research synthesis (Freetext)<br>e) None	Adapted from Page et al., 2016, 1-e1	PRISMA	Title

Introduction

ID	PRISMA Item	Text	Source	Category	Transparency Domain
03.01	Rationale	Did the author(s) describe the current state of knowledge and any uncertainties?<br>**a) Yes (1)**	New item (3-e1)	PRISMA	Introduction

		b) No			
03.02	Rationale	Did the author(s) argue why it is important to conduct the review?<br>a) **Yes (1)**<br>b) No	New item (3-e2)	PRISMA	Introduction
03.03	Rationale	Did the author(s) cite reviews that address the same or similar question(s) and explain what value their review adds?<br>*Select only one.*<br>a) **Yes (1)**<br>b) **No – stated that this is the first review of this kind (1)**<br>c) No	New item (3-e3; note that updates or replications are included in this item)	PRISMA	Introduction
03.04	Rationale	If the review includes intervention(s):<br>Did the author(s) describe how the intervention(s) work?<br>a) **Yes (1)**<br>b) No	New item (3-e4)- if	PRISMA	Introduction
04.01	Objectives	Did the author(s) state explicit objectives, research questions, or hypotheses? (select all that apply)<br>*Objectives: Study aims expressed as a sentence (e.g., "The aim of this study was to synthesize effect sizes on the association between paternal sensitivity and child externalizing adjustment problems")*	New item (4-e1); note that adherence to specific framework is not necessary here to accommodate	PRISMA	Introduction

		Research questions: Study aims expressed as a question to be answered through the synthesis (e.g., "What is the magnitude of the association between paternal sensitivity and child externalizing adjustment problems?")<br>*Hypotheses: Clear hypotheses about the presence/absence of a significant effect, and/or its magnitude, and/or direction (e.g., "We hypothesized that the summary effect of the association between paternal sensitivity and child externalizing adjustment problems is significant at p < .05 (two-tailed) and r > .10.")*<br>a) **Yes – Objectives**<br>b) **Yes – Research questions**<br>c) **Yes – Hypotheses**<br>d) No – None of the above<br><br>**Scoring:** Score 1 if at least one option from a) to c) is selected.	for breadth of sample). Note that there will be no variance given our inclusion criteria, but we opted to keep it in for additional descriptive information		
04.02	Objectives	If the review includes intervention(s):<br>Was the PICO framework (or one of its variants) used to frame the research O/Q/H?<br>**a) Yes (1)**<br>b) No	New item (4-e2)- if	PRISMA; Note that this is a blend of essential items 1 and 2 for intervention studies only. Given the possibly broad scope of our	Introduction

				sample, we chose not to code this for other types of reviews than those assessing intervention effects, where formulation frameworks are popular.	

Eligibility Criteria

ID	PRISMA Item	Text	Source	Category	Transparency Domain
05.01	Eligibility Criteria	Did the author(s) specify any eligibility criteria for the following components of the review question? (Select all that apply)<br>*It suffices that criteria for this group were defined, no judgment about reproducibility of their content.*<br>*For SRs assessing associations between two variables, one counts as the intervention/exposure (i.e., the independent) variable and one as the outcome (i.e., dependent) variable.*<br>*For SRs assessing prevalence rates, only participants and outcomes are applicable.*<br>a) **Participants**<br>b) *If intervention or association:* **Interventions/Exposures**	Nguyen et al., 2022; 5-e1	PRISMA	Eligibility Criteria

		c) *If intervention:* **Comparators**<br>d) **Outcomes**<br><br>**Scoring for Transparency Score:** Number of reported criteria / Maximum possible number of reported criteria (e.g., for an intervention study with P, I, O reported [but not C], a score of ¾ = 0.75 would be assigned)			
05.02	Eligibility Criteria	Did the author(s) make a statement regarding eligibility of studies based on study design?<br>a) **Yes**<br>b) No<br><br>**Scoring:** 0.25 points for yes	Nguyen et al., 2022; 5-e2	PRISMA	Eligibility Criteria
05.03	Eligibility Criteria	Did the author(s) make a statement regarding eligibility of studies based on their publication status?<br>*Published studies include: Studies published in peer-reviewed and non peer-reviewed journals, as book chapters or books*<br>*Unpublished studies include: Preprints, graduate theses (e.g., doctorate or master theses), working papers, unpublished manuscripts, all other research reports*<br>a) **Yes - author(s) stated both published and unpublished studies were eligible for inclusion (or that no restrictions on publication status were imposed)**	Adapted from Page et al., 2016; 5-e2	PRISMA	Eligibility Criteria

		b) **Yes - author(s) stated only published studies were eligible for inclusion**<br>c) **Yes - author(s) stated only unpublished studies were eligible for inclusion**<br>d) No such statement was made<br><br>**Scoring:** 0.25 points for yes			
05.04	Eligibility Criteria	Did the author(s) make a statement regarding eligibility of studies based on language of publication?<br>a) **Yes**<br>b) No<br><br>**Scoring:** 0.25 points for yes	Page et al., 2016; 5-e2	PRISMA	Eligibility Criteria
05.05	Eligibility Criteria	If Yes:<br>What languages were reported as being eligible for inclusion in this review?<br>a) English only<br>b) Only language(s) other than English<br>c) Mixed: English and (a) specific language(s) other than English<br>d) All languages were considered - no restrictions reported	Adapted from Page et al., 2016	Qualification	
05.06	Eligibility Criteria	Did the author(s) make a statement regarding eligibility of studies based on date of publication?<br>a) **Yes – reported restrictions regarding publication date**	New item; 5-e2	PRISMA	Eligibility Criteria

		b) **Yes – reported including all results regardless of publication date**<br>c) No<br><br>**Scoring:** 0.25 points for yes			
05.07	Eligibility Criteria	Are the eligibility criteria in line with the study objectives?<br>*Check if PICOS-related information reported within O/Q/H is reproduced within eligibility criteria. Code NA if no study O/Q/H were reported.*<br>a) **Yes (1)**<br>b) No	New item; 5-e4;	PRISMA; Note that essential element 3 was not coded because it is impossible to know without accessing primary studies. Note that we broadened the scope of this essential item (4) to incorporate other study types than intervention studies.	Eligibility Criteria

Systematic Search Procedure (Methods)

Note that items for PRISMA items #6 and #7 were condensed and merged with PRISMA-S, thus there is no 1:1 correspondence with essential items.

Supplement 3- Codebook

ID	PRISMA Item	Text	Source	Category	Transparency Domain
06.01	Information Sources and Methods	Did the author(s) search bibliographic databases? *If yes: proceed to 06.02 databases*<br>a) Yes<br>b) No	New item	Filter	
06.02	Information Sources and Methods	In a separate spreadsheet for each study ("06.02 DATABASES"):<br><br>06.02.01 List all database(s) and platform(s) that were searched **(1)** and report <u>for each source</u>:<br><br>06.02.02 Date of last search, *format YYYY-MM-DD.*<br><br>06.02.03 Was the search logic that was used to search each of these sources reported?<br>**a) Full Boolean search logic was reported (1)**<br>b) Only main index terms (e.g., MeSH terms) and/or key words without Boolean logic<br>c) Author(s) did not report any search strategy or search terms<br><br>06.02.04 Was the number of records identified reported?<br>a) **Yes (1)**<br>b) No	Adapted from Rethlefsen et al., 2023 and Nguyen et al., 2022, if	PRISMA/ PRISMA-S (Items 1,2, 15)	Systematic Search Procedure

		06.02.05 Were the dates of coverage reported?<br>*Note that "from inception" counts if also the last date of the search is reported.*<br>a) **Yes (1)**<br>b) No<br><br>Scoring: **Max. 4 points** (if all databases and platforms listed, including full Boolean search logic, number of records, and date(s) of coverage			
06.03	Information Sources and Methods	If the author(s) reported searching one or several trial registers (e.g., ClinicalTrials.gov, WHO International Clinical Trials Registry Platform), did they report: registry name, citation/link, date restrictions, date searched?<br>a) **Yes (1)**<br>b) Partially (0.5)<br>c) No	Adapted from Nguyen et al., 2022, if	PRISMA/PRISMA-S (3)	Systematic Search Procedure
06.04	Information Sources and Methods	If the author(s) reported searching any other electronic source (e.g., Google Scholar, organization websites, journal websites, preprint servers), did they report: website name, URL, search date?<br>a) **Yes (1)**<br>b) Partially<br>c) No	Adapted from Nguyen et al., 2022, if	PRISMA-S (4)	Systematic Search Procedure

06.05	Information Sources and Methods	If the author(s) contacted manufacturers or organizations, did they specify each source?<br>a) **Yes (1)**<br>b) Partially<br>c) No	New item, if	PRISMA	Systematic Search Procedure
06.06	Information Sources and Methods	If the author(s) contacted individuals to identify studies, was the type of individuals specified? (e.g., researchers in the field)<br>a) **Yes (1)**<br>b) Partially<br>c) No	New item, if	PRISMA	Systematic Search Procedure
06.07	Information Sources and Methods	If the authors(s) searched journals or conference proceedings, did they specify the names of each source, the dates covered and how they were searched (such as handsearching or browsing online)?<br>a) **Yes**<br>b) Partially<br>c) No	New item, if	PRISMA	Systematic Search Procedure
06.08	Information Sources and Methods	Did the author(s) report using backward or forward search strategies? (Select all that apply)<br>*Many terms are used to describe these strategies ("citation chasing", "pearl growing", "citation mining" etc). Code based on content of the strategy, not based on names.*<br>*Backward search: Reference lists of included articles or key publications (e.g., other systematic*	New item	Filter	

		reviews) are searched for further eligible publications ("Whom do they cite?" – references were published prior to eligible study)<br>*Forward search: References are checked that cite an eligible study ("Who cites them?" – references were published after eligible study)*<br>a) Yes – backward search<br>b) Yes – forward search<br>c) Yes – none used<br>d) Not reported			
06.09	Information Sources and Methods	If Yes – backward search:<br>Did the author(s) report how backward searches were conducted in sufficient detail to enable replication? I.e., did they specify where they conducted the backward searches (e.g., via hand-searching of all references in the publications or via a database, such as Scopus) and cited articles where references were screened?<br>*Note that if all studies eligible for inclusion were searched, it is not necessary to cite each study.*<br>a) **Yes (1)**<br>b) No	New item, if	PRISMA-S (5)	Systematic Search Procedure
06.10	Information Sources and Methods	If Yes – forward search:<br>Did the author(s) report how forward searches were conducted in sufficient detail to enable replication? I.e., did they specify the database(s) in which they conducted the forward searches, the exact search date(s), and the "base" articles?	New item, if	PRISMA-S (5)	Systematic Search Procedure

		Note that if all studies eligible for inclusion were searched, it is not necessary to cite each study.<br>a) **Yes (1)**<br>b) No			
07.01	Search Strategies	Did the author(s) report whether search limits or filters were used?<br>a) **Yes – search limits/filters used (1)**<br>b) **Yes – no search limits/filters used (1)**<br>c) No – not reported	Adapted from Lopez-Nicolas et al., 2022	PRISMA-S (9, 10)	Systematic Search Procedure
07.02	Search Strategies	If Yes:<br>Did the author(s) provide justification for the use of the limits applied to the search(es)?<br>*Note: See PRISMA-S p.11 for details if needed.*<br>a) **Yes (1)**<br>b) No	New item, if	PRISMA-S (9, 10)	Systematic Search Procedure
07.03	Search Strategies	How did the author(s) arrive at their search terms? (select all that apply)<br>*Code "expert opinion" when they indicated they thought of the search terms themselves.*<br>*Code "key literature" when they surveyed the literature in the field for possible terms. This is not the same as adapting search terms from existing previous systematic reviews. Code "not cited" for search terms from previous systematic reviews, when they did not specify from which systematic reviews these terms were taken.*	New item	PRISMA-S (11)	Systematic Search Procedure

		a) **Search terms from previous systematic reviews (cited)**<br>b) Search terms from previous systematic reviews (not cited)<br>c) **Expert opinion**<br>d) **Key literature**<br>e) **Other: Freetext**<br>f) Not reported<br><br>**Scoring**: 1 if at least one from a), c), d), e)			
07.04	Search Strategies	Did the author(s) report updating their search?<br>*If author(s) state that search was run once, code "Yes – no update".*<br>a) Yes – full search<br>b) Yes – only selected information sources<br>c) Yes – no update<br>d) No – not reported	New item	Filter	
07.05	Search Strategies	If Yes:<br>Did the author(s) report the method used to update the search(es)? (e.g., rerunning searches, email alert)<br>a) **Yes (1)**<br>b) No	New item, if	PRISMA-S (12)	Systematic Search Procedure
07.06	Search Strategies	Was the search strategy peer-reviewed?<br>*In PRISMA-S, this refers to the peer review of search strategies prior to executing the search. Peer review is used to identify errors, missing keywords or*	New item	Filter	

		subject headings, and other issues within a search strategy. One commonly used tool for search strategy peer review is the Peer Review of Electronic Search Strategies (PRESS) Guideline. Code "yes – it was peer-reviewed by another researcher" (both options), when another researcher not involved in the initial search string development validated/checked the search string.<br>a) Yes – it was peer-reviewed by another researcher using a formal tool (e.g., PRESS)<br>b) Yes – it was peer-reviewed by another researcher (without formal tool)<br>c) Yes – it was not peer-reviewed<br>d) No – not reported			
07.09		If the search strategy was peer-reviewed: Did the author(s) report any details on the peer-review process and/or the tool used?<br>**a) Yes (1)**<br>b) No	New item, if	PRISMA-S (14)	Systematic Search Procedure
07.07	Search Strategies	If the search strategy was validated (e.g., identification of a set of clearly eligible studies): Did the author(s) report details on this validation procedure and the validation set of studies?<br>a) **Yes (1)**<br>b) No	New item, if	PRISMA	Systematic Search Procedure

07.08	Search Strategies	If more than one bibliographic database was searched: Did the author(s) report the process (including any software) used to deduplicate records?<br>a) **Yes (1)**<br>b) No	New item, if	PRISMA-S (16)	Systematic Search Procedure
08.01	Selection Process	How many reviewers (humans) were involved in the screening process?<br>*Code NR if not reported. Note that this is just the overall number of people, even if one person only screened a subset of records (e.g., for reliability calculations). Check CREDIT-statement if not mentioned within text.*<br><br>[Free text]<br><br>**Scoring**: Score 0.5 if only one reviewer, score 1/3 in all other valid cases, score 0 if NR.	New item, 8-e1	PRISMA	Systematic Search Procedure
08.09	Selection Process	If more than one screener/screening round (by the same screener): Did the author(s) report how they resolved discrepancies between screeners?<br>a) **Yes**<br>b) No<br><br>**Scoring**: Score 1/3 points	New item, 8-e1	PRISMA	Systematic Search Procedure

08.02	Selection Process	Did the author(s) report whether they contacted (or attempted to contact) corresponding author(s) of included studies for any unpublished data (e.g., unreported outcomes, unreported information required for the screening process)?<br>a) **Yes - Review author(s) reported that they contacted (or attempted to contact) author(s) of included studies (1)**<br>b) **Yes - Review author(s) reported that they DID NOT contact author(s) of included studies (1)**<br>c) No such statement was made	Adapted from Page et al., 2016, 8-e2	PRISMA	Systematic Search Procedure
08.03	Selection Process	If articles required translation into another language for data collection:<br>Did the author(s) report how these articles were translated?<br>a) **Yes (1)**<br>b) No	New item, 8-e3	PRISMA	Systematic Search Procedure
08.04	Selection Process	What method of study screening did the author(s) report using?<br>a) **All titles/abstracts and full text articles were screened by at least two reviewers independently**<br>b) **All titles/abstracts and full text articles were screened using liberal acceleration (i.e. more than one reviewer needed to independently exclude a record, but only one reviewer needed to include a record)**	Adapted from Page et al., 2016, 8-e1	PRISMA	Systematic Search Procedure

		c) **All titles/abstracts and full text articles were screened by one reviewer, and one or more other reviewer(s) screened a sample of records**<br>d) **All titles/abstracts and full text articles were screened by only one reviewer once**<br>e) **All titles/abstracts and full text articles were screened by only one reviewer more than once**<br>f) **Different method applied to titles/abstracts and full text articles (e.g., titles/abstracts screened using liberal acceleration, full text articles screened by two reviewers independently)**<br>g) **More than one reviewer screened records for eligibility, but author(s) did not specify whether this method was applied independently to both titles/abstracts AND full text articles**<br>h) **All identified studies screened by one author and by an automation tool (e.g., machine learning classifier)**<br>i) Not reported<br>j) **Other (please specify): Freetext**<br><br>Scoring: Score 0.5 if only one reviewer, score 1/3 in all other cases.			

08.05	Selection Process	If records were screened more than once: Was there any measure of rater agreement reported?<br>a) **Yes, for all records (1)**<br>b) **Yes, for a subset of records (1)**<br>c) No, not reported	New item, NEW1 - if	Added (not explicitly reported within PRISMA)	Systematic Search Procedure
08.06	Selection Process	If a rater agreement was reported for the screening process, what was its metric?<br>a) Percentage agreement<br>b) Kappa (unadjusted)<br>c) Kappa (adjusted, e.g., Brennan-Prediger, also called PABAK)<br>d) Other: Freetext	New item	For use within AMSTAR-2	
08.07	Selection Process	If a rater agreement was reported for the screening process, what was its value? Freetext<br>*Report percentage agreement as 0-100 and Kappa from 0-1. Report other metrics as reported. If there is more than one value, report the lowest one.*	New item	For use within AMSTAR-2	
08.08	Selection Process	Did the author(s) report using any automation tool(s) in the screening process?<br>*Automation tool: "Here, we understand automation as a repeatable computerized method that performs a task normally executed by the researchers or that aids in their decision-making process" (Altena et al., 2019)*<br>a) Yes – they used (an) automation tool(s)<br>b) Yes – they reported they did not use (an) automation tool(s)	New item	Filter	

		c) No – not reported			

Systematic Search Procedure (Results)

ID	PRISMA Item	Text	Source	Category	Transparency Domain
16.01	Study selection	Did the author(s) report the flow of study selection, including (a) number of records from each database (if applicable), (b) number of records screened (if applicable), (c) number of records assessed in full (if applicable), (d) number of records included in review?<br>a) **Yes (a-d was reported)**<br>b) Partially – reporting some flow (some points from a-d were reported)<br>c) Partially – reporting no flow (only d was reported)<br>d) No<br><br>**Scoring**: 1 point for a), 0.5 for b), 0.25 for c)	New item, 16a-e1	PRISMA	Systematic Search Procedure
16.03	Study selection	If the systematic review is an update of a systematic review: Did the author(s) make a clear differentiation between their search and selection process for the current review and studies that were included in the previous systematic review?<br>a) **Yes (1)**<br>b) No	New item, 16a-e2	PRISMA	Systematic Search Procedure

16.02	Study selection	Did the author(s) cite studies that might appear to meet inclusion criteria, but were excluded at fulltext stage?<br>a) **Yes, a reference and reason for every excluded article was provided (1)**<br>b) No, only a list of reasons (with or without number of studies) was provided, but not on a per-study basis<br>c) No – not reported	Adapted from Sandoval-Lentisco et al., 2024, 16b-e1	PRISMA	Systematic Search Procedure

Data Collection (Methods)

**ID**	**PRISMA Item**	**Text**	**Source**	**Category**	**Transparency Domain**
09.01	Data Collection Process	How many reviewers (humans) were involved in the data collection process? Freetext - Code NR if not reported. *Note that this is just the overall number of people, even if one person only screened a subset of records (e.g., for reliability calculations).*<br><br>**Scoring:** Assigned 0 points in case NR is coded; assign 0.5 in case of 1 coder, assign 1/3 in case of more than one coder.	New item (9-e1)	PRISMA	Data Collection
09.12	Data Collection Process	If more than one coder/coding round (by the same coder): Did the author(s) report how they resolved discrepancies between coders?	New item (9-e1)	PRISMA	Data Collection

		c) **Yes**<br>d) No<br><br>**Scoring**: Score 1/3 points			
09.02	Data Collection Process	Did the author(s) report whether they contacted (or attempted to contact) corresponding author(s) of included studies for any unpublished data (e.g., unreported outcomes, unreported information required for the data collection process)?<br>a) **Yes - Review author(s) reported that they contacted (or attempted to contact) author(s) of included studies (1)**<br>b) **Yes - Review author(s) reported that they DID NOT contact author(s) of included studies (1)**<br>c) No such statement was made<br>d) Not applicable (e.g., no studies eligible for contacting author[s] identified)	Adapted from Page et al., 2016 (9-e2)	PRISMA	Data Collection
09.03	Data Collection Process	How was the data collection process described?<br>a) **More than one coder coded all included studies independently (i.e., each study was coded at least twice by two different people)**<br>b) **More than one coder coded a subset of all included studies independently (i.e., each study was coded at least twice by two different people) and remaining studies were coded by one coder**	New item (9-e1)	PRISMA	Data Collection

		**IF YES TO ITEM ABOVE: % of studies coded more than once: Freetext**<br>c) **Coding of all studies was done by one coder and was validated (but not independently coded) by at least one other coder**<br>d) **One coder coded all included studies once**<br>e) **One coder coded all included studies (at least) twice**<br>f) **Other, please specify: Freetext**<br>g) Not reported<br><br>**Scoring**: Score 0.5 if 1 coder, score 1/3 in all other cases			
09.04	Data Collection Process	If variables were coded more than once: Was there any measure of rater agreement reported?<br>a) **Yes, for all variables separately (1)**<br>b) **Yes, in the form of summary measures (e.g., mean, SD, range) (1)**<br>c) No, not reported	New item (9-NEW1-if)	PRISMA (i.e., transparency-related)	Data Collection
09.05	Data Collection Process	If a rater agreement was reported for the coding process, what was its metric? (select all that apply)<br>a) Percentage agreement<br>b) Kappa (unadjusted)<br>c) Kappa (adjusted, e.g., Brennan-Prediger, also called PABAK)	New item	Qualification	

		d) Other: Freetext			
09.06	Data Collection Process	If a rater agreement was reported for the coding process, what was its value?<br>Freetext - *Report percentage agreement as 0-100 and Kappa from 0-1. Report other metrics as reported.*	New item	To be used within AMSTAR-2	
09.07	Data Collection Process	Did the author(s) report using any automation tool(s) in the data collection process?<br>*Note: Using software to extract data from figures also counts as an automation tool for our purposes.*<br>a) Yes – they used (an) automation tool(s)<br>b) Yes – they reported they did not use (an) automation tool(s)<br>c) No – not reported	New item	Filter	
09.09	Data Collection Process	If articles required translation into another language for data collection:<br>Did the author(s) report how these articles were translated?<br>a) **Yes (1)**<br>b) No	New item (9-e3)-if	PRISMA	Data Collection
09.10	Data Collection Process	If software was used to extract data from figures (e.g., PlotDigitizer):<br>Did the author(s) specify which software was used?<br>a) **Yes (1)**<br>b) No	New item (9-e4)-if	PRISMA	Data Collection

09.11	Data Collection Process	If decision rules were used to select data from multiple reports corresponding to a study:<br>Did the author(s) report the rules and steps in the decision process and how inconsistencies were resolved?<br>*Conducting a three-level meta-analysis counts as addressing dependency.*<br>a) **Yes (1)**<br>b) No	New item (9-e5)-if	PRISMA	Data Collection
10.01	Data Items	Did the author(s) list all variables for which data were sought?<br>a) **Yes – Variables including operationalizations/categories listed (1)**<br>b) **Yes – Variables listed but not operationalizations or categories (0.5)**<br>c) No	Lopez-Nicolas et al., 2022 (10-NEW1)	PRISMA (note that 10a and 10b were merged and essential items where information from primary studies would have been necessary were dropped)	Data Collection
10.03	Data Items	10.03.01:<br>Did the author(s) specify the timeframe of measurement for the outcome variables?<br>a) **Yes - Methods: in eligibility criteria**<br>b) **Yes - Methods: in data items**<br>c) Yes - Results: e.g., table with characteristics of individual studies<br>d) No	New item (10-NEW3)	PRISMA (note that 10a and 10b were merged)	Data Collection

		10.03.02:<br>IF CORRELATIONAL:<br>Did the author(s) specify the timeframe of measurement for the other variable?<br>a) **Yes - Methods: in eligibility criteria**<br>b) **Yes - Methods: in data items**<br>c) Yes - Results: e.g., table with characteristics of individual studies<br>d) No<br><br>**Scoring Note:** Yes – results is not awarded a point because this description should be made within the methods section.<br><br>**Scoring:**<br>Score 1 point for 10.03.01 if non-correlational; score 0.5 for 10.03.01 and 10.03.02 each if correlational			
10.04	Data Items	If any tool was used to inform which data to collect: Was the tool appropriately cited?<br>a) **Yes (1)**<br>b) No	New item (10-NEW4)-if	PRISMA (note that 10a and 10b were merged and essential items were information from primary studies would have been necessary were dropped)	Data Collection

Data Collection (Results)

ID	PRISMA Item	Text	Source	Category	Transparency Domain
17.01	Study characteristics	Did the author(s) cite each study and present its characteristics?<br>*The format can be a table or figure, and it can be reported in the main text, supplementary materials, or as a raw data file.*<br>a) **Yes – both**<br>b) **Yes – citation**<br>c) **Yes – key characteristics**<br>d) No<br><br>Scoring: 2 for a), 1 for b) or c)	New item (17-e1,e2)	PRISMA	Data Collection

Risk of Bias (Methods)

ID	PRISMA Item	Text	Source	Category	Transparency Domain
11.01	Study risk of bias assessment	Did the author(s) report having undertaken a risk of bias (or quality) assessment?<br>a) Yes – undertaken<br>b) No – argued why not undertaken	New item	Filter	

		c) No – not undertaken or not reported			
11.02	Study risk of bias assessment	If Yes – undertaken:<br>Did the author(s) specify the tool (validated or self-developed) to assess risk of bias or study quality?<br>a) **Yes (name and citation provided for validated tools, content provided for self-developed tools) (1)**<br>b) No	New item, 11-e1	PRISMA	Risk of Bias
11.03	Study risk of bias assessment	If a non-standardized and/or new tool (e.g., one specially developed for the review) was used to assess risk of bias:<br>Did the author(s) report the rules they applied to reach an overall judgement?<br>a) **Yes (1)**<br>b) No	New item, 11-e2 and 11-e3, if	PRISMA	Risk of Bias
11.08	Study risk of bias assessment	If adaptations to an existing tool were made:<br>Did the author(s) describe these adaptations?<br>a) **Yes (1)**<br>b) No	New item, 11-e4, if	PRISMA	Risk of Bias
11.09	Study risk of bias assessment	If a new tool was developed:<br>Did the authors make it publicly accessible?<br>c) **Yes (1)**<br>d) No	New item, 11-e5, if	PRISMA	Risk of Bias

11.04	Study risk of bias assessment	If undertaken: how many author(s) were involved in the risk of bias (or quality) assessment? *Check CREDIT-statement if not mentioned within text.*<br>a) **All included studies assessed by at least two author(s)**<br>b) **All included studies assessed by one author, with verification by another**<br>c) **All included studies assessed by only one author**<br>d) **Other (please specify): Freetext**<br>e) Not reported<br><br>**Scoring**: Score 1 d), score 0.5 if a), b)	Adapted from Nguyen et al., 2022, 11-e6	PRISMA	Risk of Bias
11.10	Study risk of bias assessment	If more than one author:<br>Did the author(s) report how discrepancies were resolved?<br>a) **Yes (0.5)**<br>b) No	New item, 11-e6, if	PRISMA	Risk of Bias
11.05	Study risk of bias assessment	If undertaken: Did the author(s) report using any automation tool(s) for assessing risk of bias?<br>a) Yes – they used (an) automation tool(s)<br>b) Yes – they reported they did not use (an) automation tool(s)<br>c) No – not reported	New item	Filter	
11.07	Study risk of bias assessment	Did the author(s) report whether they contacted (or attempted to contact) corresponding author(s) of included studies for any unpublished data (e.g.,	Adapted from Page et al., 2016, 11-e7	PRISMA	Risk of Bias

		unreported outcomes, unreported information required for the risk of bias assessment)?<br>a) **Yes - Review author(s) reported that they contacted (or attempted to contact) author(s) of included studies (1)**<br>b) **Yes - Review author(s) reported that they DID NOT contact author(s) of included studies (1)**<br>c) No such statement was made			

Risk of Bias (Results)

ID	PRISMA Item	Text	Source	Category	Transparency Domain
18.01	Risk of bias in studies	Did the author(s) present the risk of bias assessments for each included study?<br>a) **Yes (1)**<br>b) No – even though risk of bias assessment was reported<br><br>Score NA if no risk of bias assessment was conducted.	New item	PRISMA (18-e1)	Risk of Bias
18.02	Risk of bias in studies	Did the author(s) provide justification for their risk of bias assessment?<br>*This could also appear in supplementary materials.*<br>a) **Yes (1)**<br>b) No	New item	PRISMA (18-e2)	Risk of Bias

		Score NA if no risk of bias assessment was conducted.			

Effect Measures and Statistical Synthesis (Methods)

Note that no items regarding sensitivity analyses were constructed because these would have had no variance (i.e., if they were conducted to be reported, a point would be rewarded). Note that items regarding meta-regression were condensed to accommodate large variety of models.

ID	PRISMA Item	Text	Source	Category	Transparency Domain
12.01	Effect measures	What was/were the effect measure(s) for the meta-analysis/analyses? (select all that apply)<br>a) **Risk ratio**<br>b) **Odds ratio**<br>c) **Standardized mean difference (variants of d, g)**<br>d) **Correlation coefficient (Pearson r, Spearman rank correlation)**<br>e) **Proportion**<br>f) **Other (please specify): Freetext**<br>g) Not reported<br><br>**Scoring**: Score 1 if at least one from a) to f) was selected.	Adapted from Nguyen et al., 2022 and Page et al., 2018 (12-e1)	PRISMA	Statistical Synthesis
12.02	Effect measures	Did the author(s) report the formula used to compute the effect measure (e.g., computation of d	Lopez-Nicolas et al., 2022	Added (note that this is not	Statistical Synthesis

		from M, SD, n, conversion of test statistics)? *Both the formula explicitly reported in the paper and a direct reference to a manual will be accepted, as long as it includes the exact page where to find it. Code NA for correlational or prevalence studies, but do code if formulae were reported for these.*<br>a) **Yes (1)**<br>b) No – manual but not page reported<br>c) No – nothing reported	12-NEW1	included in PRISMA, but crucial for full meta-analytic reproducibility; e.g., Maassen et al., 2020)	
12.03	Effect measures	Did the author(s) state thresholds/ranges used to interpret the size of the effect(s) and the rationale? *A citation sufficiently covers the threshold/range and rationale. Code NA for prevalence studies*<br>c) **Yes (1)**<br>d) No	New item (12-e2)	PRISMA	Statistical Synthesis
12.04	Effect measures	If synthesized results were re-expressed in a different measure (e.g., for ease of interpretation): Did the author(s) report the method they used?<br>a) **Yes (1)**<br>b) No	New item (12-e3)-if	PRISMA	Statistical Synthesis
13.01	Synthesis methods	Did the author(s) describe any data conversion techniques (e.g., r-to-z) required to prepare the data for the meta-analysis? *Code only for r, OR, RR, HR, p (prevalence), means.*<br>a) **Yes – reported (1)**<br>b) **Yes – reported that no conversion was undertaken (1)**	New item (13b-e1)	PRISMA (Note that 13a and 13c were not included due to necessary comparison with primary studies	Statistical Synthesis

		c) No – not reported		[13a], or difficulty to assess which chosen display formats are standard enough to not warrant explanation [13c]. Note that 13b was only coded with regard to effect size conversion for meta-analysis, not for information where comparison with primary studies would have been necessary)	
13.09	Synthesis methods	Which statistical software was used to perform meta-analyses? (select all that apply)<br>a) **R**<br>b) **Stata**<br>c) **RevMan**<br>d) **Comprehensive Meta-Analysis (CMA)**<br>e) **SAS**<br>f) **SPSS**<br>g) **JASP**	Adapted from Nguyen et al., 2022; 13d-e1	PRISMA	Statistical Synthesis

		h) **Python**<br>i) **Other (please specify): Freetext**<br>j) Not reported<br><br>If not R, Stata, Python: 1 point<br>If R, Stata, Python: 0.5 points			
13.10	Synthesis methods	If R, Stata or Python: Did the author(s) report which statistical package(s) were used (e.g., metan in Stata, metafor in R) and the version number of the package(s)?<br>a) **Both the statistical package(s) and software version number(s) were reported**<br>b) **Only the statistical package(s) were reported**<br>c) **Only the software version number(s) were reported**<br>d) Neither were reported<br><br>**Scoring:**<br>If a) score 0.5<br>If b) or c): score 0.25 each	Nguyen et al., 2022; 13d-e1-if	PRISMA	Statistical Synthesis
13.02	Synthesis methods	Did the author(s) specify the meta-analysis model used (e.g., fixed-effect, random-effects)? *(Note: such information may appear in the Methods section or on the forest plot)*<br>a) **Yes**<br>b) No	Nguyen et al. 2022; 13d-e3.1	PRISMA	Statistical Synthesis

		**Score** 1/3 points			
13.03	Synthesis methods	If yes: What meta-analytic model was used?<br>a) Fixed effects model<br>b) Equal effects model<br>c) Random effects model (two levels)<br>d) Random effects model (three or more levels)<br>e) Other [FREETEXT]	New item	Qualification	
13.05	Synthesis methods	Did the author(s) specify the meta-analysis weighting method used (e.g., Mantel-Haenszel, inverse-variance)? *(Note: such information may appear in the Methods section or on the forest plot)*<br>a) **Yes**<br>b) No<br><br>**Score** 1/3 points	Nguyen et al., 2022; 13d-e3.2	PRISMA	Statistical Synthesis
13.06	Synthesis methods	If Yes:<br>Which weighting method was used?<br>a) Inverse variance<br>b) Mantel-Haenszel<br>c) Peto<br>d) Sample size<br>e) Other, please specify: Freetext	New item	Qualification	
13.13	Synthesis methods	Which methods were used to identify or quantify heterogeneity? Select all that apply.<br>a) **Visual inspection of results**	New item; 13d-e3.3	PRISMA	Statistical Synthesis

		b) **A formal test for heterogeneity (e.g., "the Q test")**<br>c) **I^2**<br>d) **Tau^2 (for three-level models: also $omega^2$)**<br>e) **Prediction intervals**<br>f) **Other, please specify: Freetext**<br>g) Not reported<br><br>**Score** 1/3 points			
13.07	Synthesis methods	If random effects meta-analysis: Did the author(s) specify the estimator for the variance component(s) (e.g., DerSimonian and Laird, restricted maximum likelihood (REML)?<br>*(Note: Such information may appear in the Methods section or on the forest plot. Only answer as "Yes" if the estimator was reported explicitly. Answer as "No" if the estimator was not reported explicitly, even if you can guess the estimator from the software used [e.g., because the software implements only one option])*<br>a) **Yes**<br>b) No<br><br>**Score** 0.5 points	Nguyen et al., 2022; 13d-e4.1-if	PRISMA	Statistical Synthesis
13.08	Synthesis methods	If Yes:<br>Which estimator was used?<br>a) REML<br>b) ML	New item	Qualification	

		c) DerSimonian-Laird (also called “method of moments estimator”)<br>d) Other, please specify: Freetext			
13.04	Synthesis methods	If random effects model: Which method was used to calculate the confidence interval for the summary effect?<br>**a) Wald-typed**<br>**b) Hartung-Knapp-Sidik-Jonkman**<br>**c) Other – Freetext**<br>d) Not reported<br><br>**Score** 0.5 points	New item; 13d-e4.2-if	PRISMA	Statistical Synthesis
13.11	Synthesis methods	Did the author(s) describe any methods used to deal with possible dependent effect sizes? Either through the selection of a single effect per primary study (by random selection, decision rule or averaging) or by statistical treatment (multivariate meta-analysis, multi-level meta-analysis or robust estimation of variance).<br>a) **Yes (1)**<br>b) No	Lopez-Nicolas et al., 2022; 13d-e6	PRISMA (Note that this is not coded as an if item because some form of selection will have happened from primary studies)	Statistical Synthesis
13.12	Synthesis methods	If Yes:<br>If the article reported any method used to deal with dependent effect sizes, specify which ones:<br>a) Averaging (single effect size per independent study)	Sandoval-Lentisco et al., 2024	Qualification	

		b) Decision rule (single effect size per independent study)<br>c) Random selection (single effect size per independent study)<br>d) Multivariate meta-analysis (here: including more than one outcome; more than one effect size per independent study)<br>e) Three- or more-level meta-analysis (more than one effect size per independent study), also including multilevel RVE (Pustejosvky & Tipton, 2021)<br>f) Robust Variance Estimation (RVE) (more than one effect size per independent study) with cluster-robust standard errors (Hedges et al., 2010, Tipton, 2015)			
13.17	Synthesis methods	If it was not possible to conduct one or more of the planned syntheses, did the author(s) justify their decision as well as their alternative synthesis approach?<br>a) **Yes – decision**<br>b) **Yes – alternative approach**<br>c) **Yes - both**<br>d) No<br><br>**Scoring**: 1 for (c), 0.5 for (a) or (b)	New item; 13d-e.7-if	PRISMA	Statistical Synthesis
13.14	Synthesis methods	Did the author(s) report using any method to formally investigate heterogeneity among effect sizes (e.g., subgroup analysis, meta-regression)?	New item; 13e (quasi)	Filter (Note that this substitutes essential item 1	

		a) Yes – conducted<br>b) Yes – not conducted (also includes methods used to explore heterogeneity in studies/effect sizes not amenable for meta-analysis, e.g., structured tables.)<br>c) No – not reported		in 13e – if authors have used any method, they specified it (so no variance for us).	
13.15	Synthesis methods	If Yes - conducted:<br>Did the author(s) report which moderator variables, including their type (continuous; categorical) and levels (if categorical), were tested?<br>*This needs to be specified within the methods section.*<br>a) **Yes (1)**<br>b) No	New item; 13e (quasi)-if	PRISMA (Note that item 13e was boiled down to clearly stating moderator variables, types, and levels to accommodate wide range of methods and models, exploratory and confirmatory analyses, and not needing access to primary studies)	Statistical Synthesis

Effect Measures and Statistical Synthesis (Results)

ID	PRISMA Item	Text	Source	Category	Transparency Domain
19.01	Results of individual studies	Did the author(s) report the descriptives (e.g., M, SD, n) or test statistics used to compute the effect sizes (e.g., within a raw dataset)?<br>*Note that for effect sizes based on two dichotomous variables, this does also include the 2x2 cell counts. For proportions, this includes x (number of people with outcome) and n.*<br>a) **Yes**<br>b) No<br><br>**Scoring**: Code NA if only extraction of direct effect measures, e.g., correlation coefficients.	New item; 19-e1	PRISMA	Statistical Synthesis
19.02	Results of individual studies	Were effect estimates and measures of precision of each included study reported in a table, figure, or text?<br>*By "effect estimate", we mean a point estimate of the intervention effect, such as a mean difference for continuous outcomes, or a risk ratio, odds ratio or risk difference for binary outcomes. By "measure of precision", we mean a standard error or confidence interval.*<br>a) **Yes**<br>b) No	Nguyen et al., 2022; 19-e2	PRISMA (Note that last three essential items not coded due to displayment-related reporting or because of necessary comparison with primary studies.)	Statistical Synthesis

20.02	Results of syntheses	If the author(s) reported information on individual effect sizes: Did they provide the source for each effect size?<br>a) **Yes**<br>b) No	New item; 20a-e1 (quasi)-if	PRISMA (Note that this item was modified to explicitly incorporate effect size information as necessary for reproducibility)	Statistical Synthesis
20.03	Results of syntheses	Is it clear which effect size contributed to each synthesis?<br>a) **Yes**<br>b) No	New item; 20a-e2 (quasi)	PRISMA	Statistical Synthesis
20.01	Results of syntheses	Did the author(s) report results for all meta-analyses conducted? This includes the summary effect and its precision (e.g., confidence interval), and measures of statistical heterogeneity.<br>a) **Yes**<br>b) Partially – only some values reported<br>c) Partially – not for all meta-analyses reported<br>d) No<br><br>**Scoring**: 1 for a), 0.5 for b) or c)	New item; 20b-e2	PRISMA	Statistical Synthesis
20.06	Results of syntheses	If group differences or associations were assessed: Did the author(s) describe the direction of effects? *This could be explained within the methods section or within the results section for each analysis*.<br>a) **Yes (1)**	New item; 20b-e5-if	PRISMA	Statistical Synthesis

		b) No			
20.07	Results of syntheses	If a mean difference was assessed: Did the author(s) specify the unit of measurement and scale limits?<br>a) **Yes (1)**<br>b) No	New item; 20b-e6-if	PRISMA	Statistical Synthesis
20.08	Results of syntheses	If moderator analyses were conducted: Did the author(s) provide the moderator codings per effect size?<br>a) **Yes (1)**<br>b) No	New item; 20c (adapted)-if	PRISMA	Statistical Synthesis
20.04	Results of syntheses	If moderator analyses were conducted:<br>Did the author(s) report results for all moderator analyses conducted? For a meta-regression, this includes the regression coefficient (cont., cat.) or the effect sizes per group in no-intercept models (cat.), as well as the p-value or a confidence interval. For a subgroup analysis, this entails the summary effects and a measure of precision, measures of heterogeneity and the p-value of the interaction test.<br>a) **Yes**<br>b) **Partially**<br>c) No<br><br>**Score** 1 for a) and 0.5 for b)	New item; 20c (adapted)-if	PRISMA (Note that in line with methods item on meta-regressions, these items were simplified as compared to PRISMA essential items to accommodate wide variety of models.	Statistical Synthesis

20.05	Results of syntheses	If sensitivity analyses were conducted:<br>Were results of sensitivity analyses reported?<br>a) **Yes (1)**<br>b) No	New item; 20d-e1-if	PRISMA	Statistical Synthesis
20.09	Results of syntheses	If sensitivity analyses were conducted: Did the author(s) make a statement about the robustness of the main effect given the results from the sensitivity analyses?<br>a) **Yes (1)**<br>b) No	New item; 20d-e2-if	PRISMA	Statistical Synthesis

Reporting Bias Assessment (Methods)

**ID**	**PRISMA Item**	**Text**	**Source**	**Category**	**Transparency Domain**
14.01	Reporting bias assessment	Which methods did the author(s) use to assess reporting bias?<br>a) Statistical (i.e., publication bias detection methods)<br>b) Graphical (e.g., funnel plot)<br>c) Tools (e.g., checklists, scales, domain-based tools)<br>d) Other, please specify: Freetext<br>e) Not reported	New item	Filter (Note that we do not have variance on 14-e1)	
14.02	Reporting bias assessment	IF GRAPHICAL METHODS WERE USED, which one of the following:	New item	Qualification	

		Note that the plot needs to be a standalone method, i.e., a judgment about publication bias is made based by visual judgement only. If a plot is presented as part of a statistical publication bias detection method, such as a trim-and-fill procedure or a p-curve analysis, this is part of a statistical method and not coded here.<br>a) Funnel plot<br>b) Contour-enhanced funnel plot<br>c) Cumulative meta-analysis plot<br>d) Other, please specify: Freetext			
14.03	Reporting bias assessment	IF STATISTICAL METHODS WERE USED:<br>If the article stated any methods to assess publication bias, specify which ones (select all that apply and indicate whether method was only planned or actually used on the data)<br>a) Comparison of published vs. unpublished studies<br>b) (Pearson) correlation of effect size and sample size/standard error<br>c) Fail-safe N<br>d) Visual inspection of funnel plot<br>e) Trim and Fill<br>f) Egger's test (including three- or more-level variants; sometimes also called Sterne & Egger)<br>g) Begg-Mazumdar test (sometimes also called rank test)<br>h) PET-PEESE, FAT-PET-PEESE	Adapted from Sandoval-Lentisco et al., 2024	Qualification	

		i) p-curve<br>j) p-uniform, p-uniform*<br>k) Selection models<br>l) Test of excess significance<br>m) Other, please specify: Freetext			
14.11	Reporting bias assessment	IF A STATISTICAL METHOD WAS USED:<br>Did the author(s) specify the thresholds used to decide whether publication bias (as indicated by this method) was present?<br>a) **Yes (1)**<br>b) No	New item	Added for transparency – if, 14-NEW1	Reporting Bias
14.04	Reporting bias assessment	IF TOOLS WERE USED:<br>Did the author(s) specify the methodological components/domains/items of the tool?<br>a) **Yes (1)**<br>b) No	New item	PRISMA; 14-e2-if	Reporting Bias
14.05	Reporting bias assessment	IF TOOLS WERE USED:<br>If any adaptations were made, did the author(s) specify these adaptations? *No is coded when author(s) described adapting the tool, but not how.*<br>a) **Yes (1)**<br>b) No	New item	PRISMA; 14-e3-if	Reporting Bias
14.06	Reporting bias assessment	IF TOOLS WERE USED:<br>If a new tool was developed, did the author(s) describe the content and make it publicly accessible?	New item	PRISMA; 14-e4-if	Reporting Bias

		No: either content not described or not accessible or both<br>a) **Yes (1)**<br>b) No			
14.07	Reporting bias assessment	IF GRAPHICAL METHODS OR TOOLS WERE USED: Did the author(s) report how many reviewers were involved in the reporting bias assessment?<br>a) **Yes**<br>b) No<br>Scoring: 1 point if only one rater; 1/3 if more than one rater	New item	PRISMA; 14-e5-if	Reporting Bias
14.08	Reporting bias assessment	If more than one: Did the author(s) report whether they worked independently?<br>a) **Yes**<br>b) No<br><br>Scoring: 1/3 points	New item	PRISMA; 14-e5-if	Reporting Bias
14.09	Reporting bias assessment	If more than one: Did the author(s) report how discrepancies were resolved?<br>c) **Yes**<br>d) No<br>Scoring: 1/3 points	New item	PRISMA; 14-e5-if	Reporting Bias
14.10	Reporting bias assessment	IF TOOLS WERE USED:<br>Did the author(s) report any processes used to obtain or confirm relevant information from investigators?<br>a) **Yes (1)**<br>b) No	New item	PRISMA; 14-e6-if	Reporting Bias

Reporting Bias Assessment (Results)

ID	PRISMA Item	Text	Source	Category	Transparency Domain
21.02	Reporting biases	Did the author(s) present their assessments of risk of bias due to missing results (arising from reporting biases) for each synthesis?<br>a) **Yes (1)**<br>b) No	New item; 21-e1	PRISMA (Note that reporting guidance on funnel plot and funnel plot asymmetry were not coded because only for a fraction of publication bias detection methods, such guidance is provided)	Reporting Bias
21.01	Reporting biases	If a tool was used to assess reporting bias, did the author(s) report the results for each item included in the tool?<br>a) **Yes (1)**<br>b) No	New item, 21-e2	PRISMA	Reporting Bias

Certainty Assessment (Methods)

ID	PRISMA Item	Text	Source	Category	Transparency Domain
15.01	Certainty assessment	Did the author(s) report conducting any separate certainty assessment?<br>a) GRADE tool<br>b) Other published tool, please specify: Freetext<br>c) Self-developed tool<br>d) Narrative, idiosyncratic certainty assessment in separate section<br>e) No separate certainty assessment	New item	Filter	
15.02	Certainty assessment	IF TOOLS WERE USED:<br>Did the author(s) specify the tool and version used to assess certainty / confidence in the body of evidence?<br>a) **Yes – tool and version reported**<br>b) **Yes – only tool reported**<br>c) No – Not reported<br><br>**Scoring**: 1 for (a), 0.5 for (b)	New item; 15-e1	PRISMA	Certainty Assessment
15.03	Certainty assessment	IF TOOLS WERE USED<br>If any adaptations were made, did the author(s) specify these adaptations?<br>*No is coded when author(s) described adapting the tool, but not how.*<br>a) **Yes (1)**	New item, 15-e5	PRISMA	Certainty Assessment

		b) No			
15.04	Certainty assessment	IF GRADE OR OTHER TOOLS WERE USED<br>Did the author(s) specify the tool or system (and version) and give a brief explanation?<br>a) **Yes (1)**<br>b) No	New item, 15-e2-if	PRISMA	Certainty Assessment
15.05	Certainty assessment	IF SELF-DEVELOPED TOOL WAS USED<br>Did the author(s) report the factors considered (such as precision of the effect estimate, consistency of findings across studies) and the criteria used to assess each factor?<br>a) **Yes (1)**<br>b) No	New item, 15-e2-if	PRISMA	Certainty Assessment
15.06	Certainty assessment	IF SELF-DEVELOPED TOOL WAS USED<br>Did the author(s) describe the decision rules used to arrive at an overall judgment of the level of certainty (such as high, moderate, low, very low), together with the intended interpretation (or definition) of each level of certainty?<br>a) **Yes (1)**<br>b) No	New item, 15-e3-if	PRISMA	Certainty Assessment
15.07	Certainty assessment	Did the author(s) report how many reviewers were involved in the certainty assessment?<br>a) **Yes**<br>b) No	New item, 15-e6	PRISMA	Certainty Assessment

		**Score** 1 if only one coder, score 1/3 if more than one			
15.08	Certainty assessment	If more than one: Did the author(s) report whether they worked independently?<br>a) **Yes**<br>b) No	New item, 15-e6	PRISMA	Certainty Assessment
15.09	Certainty assessment	If more than one: Did the author(s) report how discrepancies were resolved?<br>a) **Yes**<br>b) No	New item, 15-e6	PRISMA	Certainty Assessment
15.10	Certainty assessment	If study investigators were contacted to obtain information, did the author(s) report what information was obtained and from which study?<br>a) **Yes (1)**<br>b) No	New item, 15-e7-if	PRISMA	Certainty Assessment
15.12	Certainty assessment	If standard phrases that incorporate certainty of evidence were used, did the author(s) report the intended interpretation of each phrase and reference to the source guidance?<br>a) **Yes (1)**<br>b) No	New item, 15-e10-if	PRISMA	Certainty Assessment

Certainty Assessment (Results)

ID	PRISMA Item	Text	Source	Category	Transparency Domain
22.01	Certainty of evidence	IF ANY CERTAINTY ASSESSMENT WAS MADE:<br>Did the authors report the overall level of certainty in the body of evidence (such as high, moderate, low, or very low) for each important outcome?<br>a) **Yes (all outcomes) (1)**<br>b) Partially (0.5)<br>c) No<br><br>**Score** 1 for a) and 0.5 for b)	New item, 20-e1	PRISMA	Certainty Assessment
22.02	Certainty of evidence	Did the author(s) provide justification/proof for their certainty assessment?<br>b) **Yes (1)**<br>c) No	New item, 20-e2 (adapted to fit all kind of certainty assessments; Note that 20-e3 is not coded)	PRISMA	Certainty Assessment

Discussion

ID	PRISMA Item	Text	Source	Category	Transparency Domain
23.01	Discussion	Did the author(s) provide an interpretation of the results in the context of other evidence?	New item, 23a-e1	PRISMA	Discussion

		a) **Yes (1)**<br>b) No			
23.02	Discussion	Did the author(s) discuss limitations of the evidence included in the review?<br>a) **Yes (1)**<br>b) No	New item, 23b-e1	PRISMA	Discussion
23.03	Discussion	Did the author(s) discuss limitations of the review processes used?<br>a) **Yes (1)**<br>b) No	New item, 23c-e1	PRISMA	Discussion
23.04	Discussion	Did the author(s) discuss implications of the results for practice, policy, or future research?<br>a) **Yes (1)**<br>b) No	New item, 23d-e1 (Note that this was condensed to one item as it is presumably based on journal focus/content)	PRISMA	Discussion

Automation Tools

**ID**	**PRISMA Item**	**Text**	**Source**	**Category**	**Transparency Domain**
08.15	Selection Process	If "Yes – they used (an) automation tool(s)":	New item	PRISMA	Automation Tools

		How was the automation tool integrated in the selection process?<br>a) **The automation tool was used to exclude records (i.e., they were excluded <u>solely</u> based on an automation tool)**<br>b) **The automation tool was used to "double-check" human decisions**<br>c) **Other, please specify: Freetext**<br>d) Unclear<br><br>**Score** 1 for a) to c)			
08.10	Selection Process	If "Yes – they used (an) automation tool(s)":<br>What kind of automation tool was used (select all that apply)?<br>*"Machine learning classifiers are statistical models that use training data to rank records according to their relevance. They can be calibrated to achieve a given level of recall, thus enabling reviewers to implement screening rules, such as eliminating records or replacing double with single screening." (PRISMA E&E)*<br>*Externally derived: Classifier training was done on an external dataset*<br>*Internally derived: Classifier training was done on records within this review*<br>a) **An externally derived machine learning classifier (e.g., Cochrane RCT Classifier)**<br>b) **An internally derived machine learning classifier**	New item	PRISMA	Automation Tools

		c) **Other, please specify: Freetext**<br>d) Unclear<br><br>**Score** 1 for a) to c)			
08.11	Selection Process	If externally or internally derived machine learning classifiers were used, was the following reported? (select all that apply)<br>a) **Software and version, and/or URL**<br>b) **Mode of usage (e.g., replacing a single screener, excluding records)**<br>c) **Details on training and validation (e.g., training sets, risk assessment of missed studies)**<br><br>**Score** 1/3 points for a) to c) each.	New item	PRISMA	Automation Tools
08.12	Selection Process	If the machine learning classifier was used to prioritize screening:<br>Did they provide details on software and screening rules?<br>*Screening rules: e.g., screening stopped altogether after a set number of irrelevant hits, screening switched from double to single screening once a pre-specified number or proportion of consecutive records was eliminated*<br>a) **Yes – details on software and screening rules specified (1)**<br>b) Yes – software but no screening rules specified (0.5)	New item, if	PRISMA	Automation Tools

		c) Yes – screening rules but no software specified (0.5)<br>d) No			
08.13	Selection Process	If the author(s) reported relying on crowdsourcing, did they provide details of the platform and selection process?<br>*Crowdsourcing: "Crowdsourcing involves recruiting (usually via the internet) a large group of individuals to contribute to a task or project, such as screening records." (PRISMA E&E). An example of crowdsourcing is the "Cochrane Crowd".*<br>*Previous "known" assessments: "Screening decisions for records that have already been manually checked can be reused to exclude the same records from being reassessed, provided the eligibility criteria are the same." (PRISMA E&E). An example is the "Known Assessments" service from Cochrane, where records that have been through Cochrane's citizens science platform, Cochrane Crowd, have received a final classification of either describing a randomized controlled trial (RCT) or not.*<br>a) **Yes (1)**<br>b) Partially (0.5)<br>c) No	New item	PRISMA	Automation Tools
08.14	Selection Process	If the author(s) reported relying on previous "known" assessments, did they describe the origin	New item	PRISMA	Automation Tools

		of these datasets and which types of records were excluded without manual screening?<br>a) **Yes (1)**<br>b) Partially (0.5)<br>c) No			
09.08	Data Collection Process	If Yes – they used (an) automation tool(s):<br>For the usage of automation tools within the data collection process, which of the following aspects was reported? (Select all that apply)<br>a) **Details on how/for what the tool was used (e.g., extraction of sentences for articles relevant for PICO criteria, extraction of data from figures)**<br>b) **Details on training of the tool**<br>c) **Details on the validation process (e.g., how was the extent of misclassified information estimated?)**<br><br>**Scoring:** 1/3 for each marked option.	New item	PRISMA	Automation Tools
11.06	Study risk of bias assessment	If Yes – they used (an) automation tool(s):<br>For the usage of automation tools within the risk of bias assessment, which of the following aspects was reported? (Select all that apply)<br>a) **Details on how/for what the tool was used (e.g., extraction of sentences for articles relevant for PICO criteria, extraction of data from figures)**<br>b) **Details on training of the tool**	New item	PRISMA	Automation Tools

		c) **Details on the validation process (e.g., how was the extent of misclassified information estimated?)**<br><br>**Scoring**: 1/3 points per selected category.			
15.11	Certainty assessment	If an automation tool was used, did the author(s) report details of the training and validation procedure?<br>a) **Yes (1)**<br>b) No	New item	PRISMA	Automation Tools
16.04	Study selection	If any automation tool(s) was/were used in the selection process: Did the author(s) specify the number of records screened/excluded by the tool? *If no PRISMA Flowchart is reported, this can also be made transparent within the text.*<br>a) **Yes (1)**<br>b) No	New item	PRISMA	Automation Tools

Open Science Practices

**ID**	**PRISMA Item**	**Text**	**Source**	**Category**	**Transparency Domain**
24.01	Registration and protocol	Did the author(s) report working from a protocol or a priori established methods? *There is no need to do an internet search for a protocol to answer this question.*	Page et al., 2016, 24a-e1/24b-1 (Note that these were combined)	PRISMA	Open Science Practices

		a) **Yes, and protocol is publicly available (author(s) cite the bibliographic reference for the published protocol in the main text)**<br>b) **Yes, and protocol is publicly available (author(s) cite a website where the protocol can be accessed)**<br>c) **Yes, but protocol is not publicly available (i.e. author(s) state that they worked from a protocol but do not report the location of the protocol, or only report that it is available on request)**<br>d) Unclear - the use of a protocol was only implied (e.g., by the term 'pre-specified'), please describe why you think a protocol was used<br>e) **No - they reported not working from a protocol**<br>f) Not reported<br><br>**Score** 1 for a), b), e), 0.5 for c)			
24.02	Registration and protocol	If Yes – protocol was used:<br>Where was it registered? (select all that apply)<br>a) PROSPERO<br>b) OSF<br>c) As a journal article<br>d) ResearchGate<br>e) INPLASY	New item	Qualification	

		f) Research Registry-Registry of Systematic Reviews/Meta-Analyses<br>g) protocols.io<br>h) Other, please specify: Freetext			
24.03	Registration and protocol	Did the author(s) report any amendments from the original protocol?<br>a) Yes – they reported amendments<br>b) Yes – they reported that no amendments were made<br>c) Not reported	New item	Filter	
24.04	Registration and protocol	If an amendment was made: Did the author(s) note the reason for the amendment?<br>**a) Yes**<br>b) No<br><br>**Scoring**: 0.5	New item, 24c-e1-if	PRISMA	Open Science Practices
24.05	Registration and protocol	If an amendment was made: Did the author(s) note the stage of the review when the amendment was made?<br>**a) Yes**<br>b) No<br><br>**Scoring**: 0.5	New item, 24c-e1-if	PRISMA	Open Science Practices
27.01	Availability of data, code,	Did the author(s) make any of the following publicly accessible, either as a supplementary file or file uploaded to a general-purpose repository (e.g.,	Adapted from Nguyen et al., 2022; 27-e1 and	PRISMA	Open Science Practices

	and other materials	Open Science Framework, Zenodo, GitHub) or institutional repository? Select all that apply. *Only select each if you are able to locate the relevant material, after searching all supplementary materials or links to shared materials)*<br>a) **Template data collection form(s)**<br>b) **File(s) containing (unprocessed) data extracted from included studies**<br>c) **File(s) containing data used in all analyses (e.g., Microsoft Excel or CSV spreadsheet, or RevMan file containing all study effect estimates included in meta-analyses)**<br>d) **Analytic code used to generate results (i.e. the sequence of commands used within a software package to manage and analyze data)**<br>e) **Other: Freetext**<br><br>**Scoring**: Score 1 for each; score maximum of four (e.g., if a-was already awarded a point, do not score e).	27-e2 (note that these were condensed because stating that materials are publicly available without stating where is not transparent)		
27.02	Availability of data, code, and other materials	If data, code, or materials are only available upon request: Did the author(s) provide contact details and describe circumstances under which materials will be shared?<br>**a) Yes – both**<br>**b) Yes – contact details**<br>**c) Yes – circumstances**<br>d) Not reported	New item, 27-e3, if	PRISMA	Open Science Practices

		**Scoring**: 1 for a) and 0.5 for b) or c)			

Disclosure Practices

**ID**	**PRISMA Item**	**Text**	**Source**	**Category**	**Transparency Domain**
25.01	Support	Did the author(s) include a statement indicating whether there were funding sources? *If funding only refers to open access funding provided by the university, code b).*<br>a) **Yes, the statement says that there was funding from a private or public organization, or both (1)**<br>b) **Yes, the statement says that no funding was provided (1)**<br>c) There is no funding statement<br>d) Other: Freetext	Adapted from Lopez-Nicolas et al., 2022, 25-e1	PRISMA	Disclosure Practices
25.02	Support	If there was funding: Did the author(s) describe the role of the funders/sponsors, including explicitly stating that the funders/sponsors had no role in the review?<br>**a) Yes (1)**<br>b) No	New item, 25-e2-if	PRISMA	
26.01	Competing interests	Did the author(s) report whether there were any conflicts of interest?	Lopez-Nicolas et al., 2022, 26-e1 and e2 (note	PRISMA	Disclosure Practices

		a) **Yes, the statement says that there are one or more conflicts of interest (1)**<br>b) **Yes, the statement says that there is no conflict of interest (1)**<br>c) There is no conflict of interest statement<br>d) Other: Freetext	that these were condensed)		
discl1	Generative AI Disclosure	Did the author(s) disclose using generative AI (e.g., ChatGPT) during the review process?<br>a) Yes – including those detailed in the automation tool section<br>b) Yes – including generative AI not detailed in the automation tool section<br>c) Yes - both<br>d) No – disclosed that not used<br>e) No such statement	New item		
