# Supplementary material for: Transparency, quality, and statistical consistency of meta-analytic systematic reviews in clinical child and adolescent psychology (2022–2024): study protocol for a meta-review
Source: Front Psychol. 2025 Jul 28;16:1535606. doi: 10.3389/fpsyg.2025.1535606 (PMC12336221; doi:10.3389/fpsyg.2025.1535606)
Supplement: Supplementary file 5 [file Supplementary_file_5.docx]

**Supplement S5: Data Management and Availability Plan**

The first author [MS] is responsible for data management. Search results from *Web of Science* will be exported as .xls, converted to .xlsx, and prepared for title and abstract screening (i.e., inserting columns for screening decisions and notes) by MS, SF, and JR. Initial interrater reliability and discrepant cases will be calculated and identified in R (via RStudio IDE, Posit), using (meta-)packages {tidyverse} (Wickham et al., 2019) and {irr} (Gamer et al., 2019) in their latest CRAN-version at this time as well as a dedicated R project. A homogenized .xlsx-sheet will serve as the basis for fulltext retrieval of all potentially eligible records. Fulltexts will be retrieved as .pdfs and .html (for statcheck-based analyses). Again, eligibility based on fulltexts will be noted via .xlsx-sheets and compared across all three authors in R, using tidyverse and irr (meta-)packages.

For coding of eligible systematic reviews, we will use our piloted coding spreadsheets (.xlsx; one sheet per systematic review) as found within Supplement 4, <https://osf.io/uy2mj>. MS will synthesize information from individual spreadsheets per coder in tidy format, such that coded items represent variables and coded reviews represent rows (one row per review) within R using the tidyverse meta-package (Wickham et al., 2019). The resulting data frames (one per coder, i.e., three overall) will be linked to calculate interrater reliability and resolve discrepant cases. A homogenized, final datasheet containing codings of general information, transparency ratings, and quality ratings will be produced as an .xslx-file. Importantly, our aim is not to highlight shortcomings of individual systematic reviews. Thus, we will identify included reviews only by an ID-number within the main text, where authors can link these back to the publication using a key provided within supplementary materials. This preserves full transparency while not highlighting individual studies within the main text.

For statcheck-based analyses, .html-files, including unique study identifiers, will be used as input. Statcheck provides a data frame with one row per analyzed document as output, which will then be linked to manually coded information (see above) in R. All further analyses will be carried out in R, using the {tidyverse} meta-package (Wickham et al., 2019) as well as {gt} (Iannone et al., 2023) and possibly {gtsummary} (Sjoberg et al., 2021). Analysis scripts will be written in .qmd documents with additional rendering as .html.

We will make the full R project used for analysis, including all data files (.rds, .csv, .xlsx), analysis scripts (.qmd, .html), and README-files (.txt) available on OSF upon first publication as a preprint (psyarxiv.org). The {renv} package (Ushey & Wickham, 2024) will be used for a reproducible analysis environment.

**References**

Gamer, M., Lemon, J., & IFPS. (2019). *irr: Various coefficients of interrater reliability and agreement (Version 0.84.1)* [Computer software]. https://CRAN.R-project.org/package=irr

Iannone, R., Cheng, J., Schloerke, B., Hughes, E., Lauer, A., & Seo, J. (2023). *gt: Easily create presentation-ready display tables*. https://CRAN.R-project.org/package=gt

Sjoberg, D., D., Whiting, K., Curry, M., Lavery, J., A., & Larmarange, J. (2021). Reproducible summary tables with the gtsummary package. *The R Journal*, *13*(1), 570. https://doi.org/10.32614/RJ-2021-053

Ushey, K., & Wickham, H. (2024). *renv: Project environments*. https://CRAN.R-project.org/package=renv

Wickham, H., Averick, M., Bryan, J., Chang, W., McGowan, L., François, R., Grolemund, G., Hayes, A., Henry, L., Hester, J., Kuhn, M., Pedersen, T., Miller, E., Bache, S., Müller, K., Ooms, J., Robinson, D., Seidel, D., Spinu, V., … Yutani, H. (2019). Welcome to the tidyverse. *Journal of Open Source Software*, *4*(43), 1686. https://doi.org/10.21105/joss.01686
